# Supplementary material for: Polyunsaturated fatty acid production by Yarrowia lipolytica employing designed myxobacterial PUFA synthases
Source: Nat Commun. 2019 Sep 6;10:4055. doi: 10.1038/s41467-019-12025-8 (PMC6731297; doi:10.1038/s41467-019-12025-8)
Supplement: Supplementary file 1 — Supplementary Information [file 41467_2019_12025_MOESM1_ESM.pdf]

Supplementary Information

Polyunsaturated fatty acid production by *Yarrowia lipolytica* employing designed myxobacterial PUFA synthases

Gemperlein et al.

Supplementary Notes

Besides the first codon optimization strategy, which was applied for the design of the gene sequences in gene cluster C1_V1, we aimed at testing the effect of an alternative gene optimization approach on recombinant LC-PUFA production in *Y. lipolytica*. Thereby, the transfer of the course of the codon usage along the message from the prokaryotic donor to the eukaryotic acceptor was regarded as the simplest formalism of codon adaptation with a high plausibility. Hence, the sequence of the *pfa* gene cluster version C1_V2 (**Fig. 2a**), encoding the DPA/DHA-type PUFA synthase from *A. fasciculatus*, was designed towards a formally improved translational elongation via a "codon harmonization" resembling strategy, where the course of the local codon adaptation index along the native mRNA of the source species was imitated in the target host species (e.g., a less well-adapted and thus assumed to be rather slowly translated region in the native sequence was replaced by a rarer one in the target and *vice versa*). Especially, rare codons should be conserved, as it has been shown that they have an important role for the production of functional proteins, potentially in the regulation of the rate of protein synthesis and of the earliest steps of protein folding[1] (all details to the sequence design can be found in **Supplementary Figs. 9-11, 13-19 and Supplementary Tables 4-8, 14-19**). Four building blocks containing the three *pfa* coding sequences as well as the *ppt* coding sequence flanked by hp4d promoters and *LIP2* terminators, plus intergenic linker sequences were synthesized, assembled, and finally cloned into plasmid pKG2-PIS. The yeast cassette of the resulting plasmid pAf7 was transferred into *Y. lipolytica* Po1h and selected clones were screened for correct integration of the transgenes into the preferred integration site (YALI0_C05907g). *Y. lipolytica* Po1h::Af7 was cultivated in triplicates as described above, followed by isolation and analysis of the FAMEs. Production of DHA at a concentration of 10.6 % of TFAs, 88.7 mg L^{-1}, and 12.2 mg g^{-1} CDW and some minor amounts of *n*-6 DPA and *n*-3 DPA were achieved (**Fig. 2c and Supplementary Table 9**). Interestingly, *Y. lipolytica* Po1h::Af7 produces DHA, *n*-6 DPA, and *n*-3 DPA in comparable

amounts as *Y. lipolytica* Po1h::Af4, containing gene cluster C1_V1. The same LC-PUFA yields obtained with two artificial *pfa* gene clusters differing in their codon usage modulation indicate possible bottlenecks within the LC-PUFA production pipeline in the cells, which need finally to be identified and eliminated in order to further improve the production yield. We first aimed at comparing the amount of Pfa proteins in the cells of strain *Y. lipolytica* Po1h::Af4 and of strain *Y. lipolytica* Po1h::Af7. A straightforward experiment for detection of Pfa proteins within the cells represents the fluorescence measurement upon the fusion of the fluorescent protein mCherry at the C-terminus of the Pfa proteins. In this experiment, all produced Pfa proteins that are not truncated caused by aborted translation should account for the fluorescence signal. Only proteins Pfa2 and Pfa3 were tagged with mCherry, since translational elongation is especially critical in case of the long mRNAs of the multifunctional PUFA synthase genes *pfa2* and *pfa3*. Four expression constructs were cloned by fusion of the open reading frame of gene *mcherry* with the last sense codon at the 3' end of either gene *pfa2* or gene *pfa3* of the two different synthetic *pfa* gene clusters in a PCR-based approach. *Y. lipolytica* Po1h was transformed with the yeast cassette of any of the final expression constructs pAf4-*pfa2-mcherry*, pAf4-*pfa3-mcherry*, pAf7-*pfa2-mcherry*, and pAf7-*pfa3-mcherry*. Two clones of *Y. lipolytica* Po1h::Af4-*pfa2-mcherry*, *Y. lipolytica* Po1h::Af4-*pfa3-mcherry, Y. lipolytica* Po1h::Af7-*pfa2-mcherry*, as well as *Y. lipolytica* Po1h::Af7-*pfa3-mcherry*, each possessing the transgenes integrated into the preferred integration site (YALI0_C05907g), were cultivated in triplicates as described above. The mCherry fluorescence signal from the liquid cultures was analyzed by fluorescence spectroscopy. Remarkably, the fluorescence signals obtained from the clones of *Y. lipolytica* Po1h::Af4-*pfa2-mcherry* were largely identical to those obtained from the clones of *Y. lipolytica* Po1h::Af7-*pfa2-mcherry*, indicating the production of comparable amounts of full-length protein Pfa2 by the two different strains. The same observation was made for the fluorescence signals received from the clones of *Y. lipolytica* Po1h::Af4-*pfa3-mcherry* and *Y. lipolytica*

Po1h::Af7-*pfa3-mcherry* (**Supplementary Fig. 19**). Possible explanations for these findings might be that translation and/or co-translational folding processes are robust and largely independent of the two codon optimization strategies or that "key codons" with special relevance for translation and/or co-translational folding exist, which are identical in the two synthetic *pfa* gene cluster versions (87.9 % of the codons in *pfa2*_V1 and *pfa2*_V2 and 86.3 % of the codons in *pfa3*_V1 and *pfa3*_V2 are identical). It is also conceivable that one of the two versions has the potential for production of higher amounts of functional proteins Pfa2 and Pfa3 but the limit is already reached with each version due to bottlenecks in biosynthesis or posttranslational modifications of Pfa2/Pfa3 or due to their degradation.

Supplementary Methods

The four building blocks of synthetic gene cluster version C1_V2 were stitched together in plasmid pACYC_BB1-4_C1_V1: Building block 1 was inserted into pACYC_BB1-4_C1_V1 via *Sda*I and *Apa*LI restriction sites, generating plasmid pACYC_BB2-4_C1_BB1_V2; building block 4 was inserted into pACYC_BB2-4_C1_BB1_V2 via *Avr*II and *Pac*I restriction sites, generating plasmid pACYC_BB2+3_C1_BB1+4_V2; building block 2 was inserted into pACYC_BB2+3_C1_BB1+4_V2 via *Apa*LI and *Acl*I restriction sites, generating plasmid pACYC_BB3_C1_BB1+2+4_V2; building block 3 was inserted into pACYC_BB3_C1_BB1+2+4_V2 via *Acl*I and *Avr*II restriction sites, generating plasmid pACYC_BB1-4_C1_V2. The backbone of plasmid pACYC_BB1-4_C1_V2 was exchanged for the backbone of plasmid pKG2-PIS via *Sda*I and *Pac*I restriction sites, yielding plasmid pAf7.

In order to monitor the expression of the artificial PUFA biosynthetic pathways version C1_V1 and C1_V2 in *Y. lipolytica*, the open reading frame of gene *mcherry* was fused with

the last sense codon at the 3' end of either gene *pfa2* or gene *pfa3* by overlap extension PCR: In case of the 3'*pfa2-mcherry* fusion, 1.1 kb of the 3' end of *pfa2* plus the 5' end of *mcherry* as overlapping sequence were amplified as the first fragment from plasmid pAf4 using primers *pfa2*_fwd and *pfa2*+*mcherry* overlap_rev in case of C1_V1 or from plasmid pAf7 using primers *pfa2*_fwd and *pfa2*+*mcherry* overlap_rev_2 in case of C1_V2. The second fragment (0.8 kb) contained the open reading frame of a synthetic *mcherry* gene plus the 3' end of *pfa2* and the 5' part of the *LIP2* terminator as overlapping sequences and was amplified from a standard cloning vector supplied by a gene synthesis company using primers *mcherry*+*pfa2* overlap_fwd and *mcherry*+*LIP2*t overlap_rev in case of C1_V1 or using primers *mcherry*+*pfa2* overlap_fwd_2 and *mcherry*+*LIP2*t overlap_rev in case of C1_V2. The *LIP2* terminator plus the 3' end of *mcherry* as overlapping sequence and parts of linker sequence 2 were amplified as the third fragment (0.2 kb) from plasmid pAf4 using primers LIP2t+*mcherry* overlap_fwd and linker 2_rev. PCR amplification of the three fragments to be spliced was performed with Phusion DNA polymerase (Thermo Scientific) under standard conditions according to the manufacturer's protocol. The reactions contained 5 % DMSO and were carried out in an Eppendorf Mastercycler under the following conditions: initial denaturation for 2 min at 98 °C; 20 cycles consisting of denaturation for 15 s at 98 °C, annealing for 20 s at 67 °C, and extension for 10 s/kb at 72 °C; and a final extension for 10 min at 72 °C. For the subsequent overlap extension PCR using primers *pfa2*_fwd and linker 2_rev, the three amplified fragments were used as templates in each case. PCR of the 1.9 kb fragments 3'*pfa2-mcherry-LIP2*t-linker 2 was performed as described for the amplification of the three fragments to be spliced. In order to generate plasmids pACYC_BB1-4_C1_V1_*pfa2-mcherry* and pACYC_BB1-4_C1_V2_*pfa2-mcherry*, the 3' end of synthetic gene *pfa2* originating from *A. fasciculatus* (SBSr002) located on plasmid pACYC_BB1-4_C1_V1 or plasmid pACYC_BB1-4_C1_V2 was replaced by the respective PCR amplicon via *Kfl*I and *Acl*I restriction sites. The backbones of plasmids pACYC_BB1-4_C1_V1_*pfa2*-

mcherry and pACYC_BB1-4_C1_V2_*pfa2-mcherry* were exchanged for the backbone of plasmid pKG2-PIS via *Sda*I and *Pac*I restriction sites, yielding plasmids pAf4-*pfa2-mcherry* and pAf7-*pfa2-mcherry*. In case of the 3'*pfa3-mcherry* fusion, 1.1 kb of the 3' end of *pfa3* plus the 5' end of *mcherry* as overlapping sequence were amplified as the first fragment from plasmid pAf4 using primers *pfa3*_fwd and *pfa3*+*mcherry* overlap_rev in case of C1_V1 or from plasmid pAf7 using primers *pfa3*_fwd and *pfa3*+*mcherry* overlap_rev_2 in case of C1_V2. The second fragment (0.8 kb) contained the open reading frame of a synthetic *mcherry* gene plus the 3' end of *pfa3* and the 5' part of the *LIP2* terminator as overlapping sequences and was amplified from a standard cloning vector supplied by a gene synthesis company using primers *mcherry*+*pfa3* overlap_fwd and *mcherry*+*LIP2*t overlap_rev in case of C1_V1 or using primers *mcherry*+*pfa3* overlap_fwd_2 and *mcherry*+*LIP2*t overlap_rev in case of C1_V2. The *LIP2* terminator plus the 3' end of *mcherry* as overlapping sequence and parts of linker sequence 3 were amplified as the third fragment (0.2 kb) from plasmid pAf4 using primers LIP2t+*mcherry* overlap_fwd and linker 3_rev. PCR amplification of the three fragments to be spliced was performed with Phusion DNA polymerase (Thermo Scientific) under standard conditions according to the manufacturer's protocol. The reactions contained 5 % DMSO and were carried out in an Eppendorf Mastercycler under the following conditions: initial denaturation for 2 min at 98 °C; 20 cycles consisting of denaturation for 15 s at 98 °C, annealing for 20 s at 67 °C, and extension for 10 s/kb at 72 °C; and a final extension for 10 min at 72 °C. For the subsequent overlap extension PCR using primers *pfa3*_fwd and linker 3_rev, the three amplified fragments were used as templates in each case. PCR of the 2.0 kb fragments 3'*pfa3-mcherry-LIP2*t-linker 3 was performed as described for the amplification of the three fragments to be spliced. In order to generate plasmid pACYC_BB1-4_C1_V1_*pfa3-mcherry*, the 3' end of synthetic gene *pfa3* originating from *A. fasciculatus* (SBSr002) located on plasmid pACYC_BB1-4_C1_V1 was replaced by the respective PCR amplicon via *Pdi*I and *Avr*II restriction sites. The backbone of plasmid pACYC_BB1-4_C1_V1_*pfa3-mcherry*

was exchanged for the backbone of plasmid pKG2-PIS via *Sda*I and *Pac*I restriction sites, yielding plasmid pAf4-*pfa3-mcherry*. Plasmid pAf7-*pfa3-mcherry* was generated by replacing the 3' end of synthetic gene *pfa3* originating from *A. fasciculatus* (SBSr002) located on plasmid pAf7 with the respective PCR amplicon via *Fse*I and *Avr*II restriction sites. Sequences of primers used in this study are listed in **Supplementary Table 11** and further information on the plasmids constructed in this study are provided in **Supplementary Table 12**.

The fluorescence of mCherry produced by yeast cells grown in liquid YNBG medium was determined using an Infinite F200 PRO fluorescence microplate reader (Tecan). Ahead of the measurements, 20 µL of the cultures were mixed with 180 µL water in a 96-well microtiter plate. For fluorescence measurements (top reading), an excitation wavelength of 550±10 nm and an emission wavelength of 580±20 nm were used. The absorbance was measured at a wavelength of 600 nm. Blank values were generated by using water. For data analysis, the obtained absorbance signals were normalized by subtracting the mean of the blank values, and the obtained fluorescence signals were normalized by subtracting the mean of the blank values followed by dividing by the normalized absorbance values.

Supplementary Discussion

The coding sequences of the artificial *pfa* gene clusters C1_V1 and C3 were designed by aiming at improved translational elongation via silent mutations, whereby the coding sequences were adapted to the synonymous codon frequencies of *Yarrowia lipolytica* CLIB122, excluding a part of the minor codons. Such a balanced codon use is supposed to be more effective than using only the preferred codons[2]. All codons below a synonymous codon fraction of 20 % in the 6448 protein coding sequences of *Y. lipolytica* were not used in the

artificial *pfa* gene clusters C1_V1 and C3 (**Supplementary Table 1**). As *Y. lipolytica* shows a higher preference for AT-rich codons as myxobacteria, the mean GC content of the coding sequences was lowered from 74 % (native *pfa* gene cluster of *Aetherobacter fasciculatus* (SBSr002)) and 71 % (native *pfa* gene cluster of *Minicystis rosea* (SBNa008)) to 62 % (artificial *pfa* gene cluster C1_V1) and 61 % (artificial *pfa* gene cluster C3), respectively. The results of the codon adaptation are shown in **Supplementary Figs. 1 and 2.** The translation rate of a certain codon depends predominantly on the concentration of its aminoacyl-tRNA isoacceptors, and it has been shown that codon frequencies are positively correlated with tRNA concentrations[3]. The relative adaptiveness scoring system implemented in the Codon Adaptation Index (CAI)[4] - where the individual codon frequencies are scaled by the highest frequency within the corresponding synonymous codon group - was intended to build in a slight gradient of ribosomal kinetics between start and stop codon of each coding DNA sequence. This aims at the enhancement of the ribosomal occupancy along the mRNA to shield the transcript from the degradosome, provided that the translation initiation rate is sufficiently high[5,6] (**Supplementary Figs. 7 and 8**). The design of the artificial *pfa* gene cluster C3_mod 5' included the optimization of the translation initiation site (TIS), concerning accessibility of the capping region[7], the Kozak sequence including the start codon[8], and the 5' region of the CDS[9], expressed as opening energies calculated with algorithms from the ViennaRNA Package[10]. The TISs from artificial *pfa* gene clusters C3 and C3_mod 5' are visualized in **Supplementary Figs. 4 and 5,** respectively. Numeric values for the selected control positions within the two artificial clusters are tabulated in **Supplementary Table 2**. Relative differences between the values for the two clusters, concerning the selected parameters, are given in **Supplementary Table 3**, and the results hint at an improvement of the TIS of cluster C3_mod 5' compared to cluster C3.

The coding sequences of the artificial *pfa* gene cluster C1_V2 were designed towards an adapted translation elongation velocity profile. In this approach, the given course and shape of

the estimated elongation speed along the mRNA is modulated, thereby recording the kinetic profile of the genes in the source organism to mimic it in the target host. The reason behind this approach is the assumption that a specific modulation of the elongation velocity is needed to prevent misfolding of the nascent polypeptide[11]. However, rather than pausing or decrease in speed, a higher velocity might enable correct protein folding[12]. There are two basic concepts: The first is "codon harmonization", where the transfer happens codon by codon according to the frequency ranking within a synonymous codon group in each of the organisms considered[13,14]. The second consists of "sliding window" approaches using different window sizes (e.g., 17, 18, 21, 25 or 30 codons wide[15-19]) and differently processed codon features, such as for example tRNA concentrations[15], tRNA gene copy numbers[17,19], or differently obtained codon frequencies[16,18]. Some of the latter techniques were used for analysis purposes only. For the transfer of the translation elongation profile, the local CAI with a window span of 19 codons was used, contrary to the 25 codon windows used for C1_V1[18]. It has been shown that the CAI is a very competitive predictor of translation efficiency in *Saccharomyces cerevisiae*[20], presumed that a subset of putatively highly expressed genes is used for calculation. Besides this, own unpublished analyses with high quality ribosome profiling data[21,22] for *S. cerevisiae* provided convincing results in this respect. Utilizing the full genomic codon table[23] to determine the codon scores for CAI calculation like it was done for the design of version C1_V1 (**Supplementary Table 1**) resembles more the approach applied in the framework of the %MinMax[16]. Presumed that the codon bias is unidirectional the correlation with translation speed should remain similar for both approaches because of the linkage with tRNA levels in both cases. To calculate the codon scores for the design of the artificial cluster C1_V2, initially a set of 92 putative highly expressed genes, predominantly coding for ribosomal proteins with lengths over 200 bp (**Supplementary Table 19)**, was chosen from the 6448 protein coding sequences of the *Y. lipolytica* CLIB122 genome (GenBank RefSeq chromosome A-F). After generating the codon

table, whereby the first 60 bp of the CDS and the last 30 bp of the CDS were omitted, the relative adaptiveness scores were calculated according to the original CAI definition (column 'w' in **Supplementary Table 15**). In the case of *A. fasciculatus* (SBSr002), the available 9638 CDS were translated according to the standard code and supplied to the KEGG BlastKOALA server (https://www.kegg.jp/blastkoala/) to obtain a collection of ribosomal proteins and elongation factors which are commonly assumed to be highly expressed. A set of 64 sequences was recognized, leading to a final collection of 57 reference genes by applying the 200 bp length filtering step (**Supplementary Table 18)**. The same procedure as for *Y. lipolytica* was used to generate the codon scores (column 'w' in **Supplementary Table 16**). For the adaptation of the synonymous codon usage fractions of the genes *pfa1*, *pfa2*, *pfa3*, and *ppt* of the artificial *pfa* gene cluster C1_V2, a target codon table was generated by cutting out a sub-cluster of the whole protein coding sequences by a maximum likelihood clustering approach. After subsequently performing the length filtering, a homogenous set of 1,609 coding sequences of *Y. lipolytica* in terms of codon usage was obtained in this way. Comparison with RNA-Seq data from the related strain *Y. lipolytica* CLIB89 showed that this fraction of the genomic CDS represents nearly 60 % of the protein coding transcriptome obtained by weighting the CDSs from the whole CLIB122 genome by the expression levels of the corresponding CLIB89 transcripts. Furthermore, the distribution of the synonymous codon fractions of this sequence set has more similarity to the calculated transcriptomics codon usage than either the genomic codon usage or the codon usage of the putative highly expressed genes from the (ribosomal) reference set. In fact, there is a gradually increasing bias towards the preferred codons from the genomic codon usage (**Supplementary Table 1**), to the automatically sub-clustered sequences (**Supplementary Table 14**), and finally to the codon usage of the reference set used for the formal CAI calculation (**Supplementary Table 15**). For comparison purposes the coding sequences of *A. fasciculatus* (SBSr002) were clustered in the same way. Results from a formal quantification of codon bias[24] regarding the

two organisms is shown in **Supplementary Table 17**. The clustering was performed by simply maximizing the raw value of the G-test statistic (column 'G' in **Supplementary Table 17)** by a variant of a stochastic hill-climbing heuristic[25]. Thereby, the expected codon counts correspond to the whole genome and the formally observed ones to a subset thereof (in analyses e.g., a preselected high expression subset[24]). For clustering, the initially random composition of the subset is varied until the algorithm has converged. For the applied threshold used to remove the putative slowest codons from the preliminary codon table, a slightly different but related procedure compared to the procedure for C1_V1 was applied. The 20 codons with the lowest relative adaptiveness values in the putative high expression set (column 'w' in **Supplementary Table 15**) were removed from the generated table. Afterwards, the table was normalized to yield the target codon fractions f_n used for optimization of cluster C1_V2. Consequently, a relative adaptiveness threshold of 0.04 was applied, following standard conventions for the CAI definition by using a ribosomal subset of coding sequences for the codon score calculation. Results of the codon adaptation of cluster C1_V2 to the target fractions f_n from **Supplementary Table 14** are shown in **Supplementary Fig. 13.** Results from the transfer of the local CAI profiles are shown in **Supplementary Figs. 15-18.** The blue line depicts the profile of the native local codon adaptation in *A. fasciculatus* (SBSr002), whereby the calculation is based on **Supplementary Table 16.** The red line depicts the transferred profile based on the codon usage of the reference set comprising the 92 putative highly expressed genes from *Y. lipolytica* CLIB122 shown in **Supplementary Table 15**. For comparison, also the profiles of the native *pfa* coding sequences from *A. fasciculatus* (SBSr002) in *Y. lipolytica* are shown in violet. The agreement of the blue and red lines in respect to the absolute CAI values is not intended but pure coincidence, because the simultaneous adaptation to the synonymous codon fractions f_n of the reduced codon set in **Supplementary Table 14** determines the absolute values on the CAI scale based on **Supplementary Table 15**. Using a larger subset of codons in the artificial codon table would

lead to smaller absolute local CAI values in the curve representing *Y. lipolytica* because lower-frequency codons, with consequently lower adaptiveness scores, would be used (note that the codon tables in **Supplementary Tables 1, 14, and 15** are similar in respect to their codon bias pattern**)**. In the case of version C1_V2, the mean GC content of the coding sequences was lowered from 74 % (native *pfa* gene cluster of *A. fasciculatus* (SBSr002)) to 63 %). Additionally, in contrast to the adaptation of the *pfa* genes from clusters C1_V1 and C3, local secondary structures were suppressed to cancel out their influence on local translation efficiency[26] (for details see **Supplementary Fig. 6)**. It is assumed that codon usage is the dominant factor, concerning the efficiency of translation elongation, at least in the absence of stable secondary structures with exceptionally low ΔG values. In **Supplementary Table 5**, the corresponding values are tabulated for the native *pfa* cluster from *A. fasciculatus* (SBSr002), the artificial *pfa* cluster V1_C1, and the artificial *pfa* cluster C1_V2. For the latter, a lower ΔG threshold of -15 kcal/mol, respecting the ensemble free energy calculated for a window of 41 nt, was applied.

For all the clusters C1_V1, C1_V2, and C3 specific sequences within the artificial genes which could be recognized as splicing signals by *Y. lipolytica*[27] were reduced to avoid splicing of the intronless pre-mRNAs. Another important factor to be considered is the metabolic cost of translational miselongation upon a frame shift. In order to terminate translation of incorrect reading frames early, the ribosome must encounter an off-frame stop codon. Furthermore, it has been shown that hidden stop codons in unused frames have a higher occurrence frequency than expected in natural sequences[28]. To keep mistranslation products as short as possible, the density of hidden stop codons was elevated. Additionally, polyadenylation signals were avoided as well as long sequence repeats[29] and homopolymeric stretches[30].

Concerning the nearly equal PUFA yield obtained with the two versions C1_V1 and C1_V2 of the artificial biosynthetic *pfa* gene clusters, an additional plausibility evaluation was

performed, including the re-analysis of the translation elongation profile by means of a modified version of the original tRNA adaptation index (tAI)[31]. The utilized tAIaa[20,32,33] (or AAtAI) is formally equivalent to the CAI but uses weighted tRNA gene copy numbers (GCN) instead of codon counts. By means of analyzing the GCN of both organisms, the tRNA sparing strategy[34] was deduced and the possible anti-codon base modifications[35] considered. Through the assumption of simple but plausible wobble interactions[36] also the uncertainties in the case of the Deltaproteobacterium *A. fasciculatus* (SBSr002) were respected[37] (**Supplementary Fig. 9**). The gene copy numbers of the direct matching tRNA isoacceptors for each of the two organisms are given in column 'GCN' of **Supplementary Table 15** for *Y. lipolytica* CLIB122 and in **Supplementary Table 16** for *A. fasciculatus* (SBSr002). Gene copy numbers for *Y. lipolytica* were downloaded from GtRNAdb[38] (http://gtrnadb.ucsc.edu/). Those for *A. fasciculatus* (SBSr002) were obtained by analyzing the genome sequence with tRNAscan-SE[39] (http://lowelab.ucsc.edu/tRNAscan-SE/). In **Supplementary Fig. 9**, the CAI and tAIaa values for each protein coding sequence of *A. fasciculatus* (SBSr002) and *Y. lipolytica* are displayed. The calculated spearman correlation coefficients are 0.933 ($p<0.001$; permutation test with 10,000 randomizations) and 0.992 ($p<0.001$; permutation test with 10,000 randomizations), respectively. Hence, it seems plausible that the CAI is a reasonable approximation of tRNA adaptation and therefore translation efficiency[20], which could be proven less speculative with ribosome profiling data, as in the case of *S. cerevisiae*[21,22]. Blue dots in the scatter plots depict the automatically clustered gene subsets for each organism, which correspond to the upper right quadrant. Using the local tAIaa with a window size of 19 codons, the approximate translation velocity profile of the *pfa2* and *pfa3* protein coding sequences from clusters C1_V1 and C1_V2 are again plotted in **Supplementary Figs. 10 and 11**, respectively. The slight gradient intended for version C1_V1 is still present, visible in the linear fit. The profiles of the local tAIaa of *pfa2* and *pfa3* from cluster version C1_V2 are similar to the local CAI in corresponding **Supplementary Figs. 15-18.** Following the same

method as for the versions C3 and C3_mod 5', the local opening energies for various control positions within the TISs of the three *pfa* genes and gene *ppt* of the artificial *pfa* gene clusters C1_V1 and C1_V2 are displayed in **Supplementary Figs. 4 and 14**, respectively. The values are summarized in **Supplementary Table 4**. Codon adaptation indices and tAIaa values of the protein coding sequences of genes *pfa1*, *pfa2*, *pfa3*, and *ppt* from the artificial biosynthetic gene clusters C1_V1 and C1_V2 and for comparison also from the native *pfa* biosynthetic gene cluster are tabulated in **Supplementary Table 6**. An offset of 20 codons at the 5' region of the CDS and 10 codons at the 3' end was applied. A summary of the translation initiation sites and the global sequence features of the CDSs, including global parameters of the local ΔG values of genes *pfa1*, *pfa2*, *pfa3*, and *ppt* from the artificial *pfa* biosynthetic gene clusters C1_V1 and C1_V2, is shown in **Supplementary Table 7.** The corresponding relative differences are tabulated in **Supplementary Table 8**. According to recent investigations, all structures with Gibbs free energy values above -18 kcal/mol within the UTR can provide decent initiation rates[40]. The global CAI and tAIaa values for version C1_V1 and version C1_V2 deviate from each other primarily due to the slightly different threshold definitions combined with the codon adaptation.

Supplementary Figures

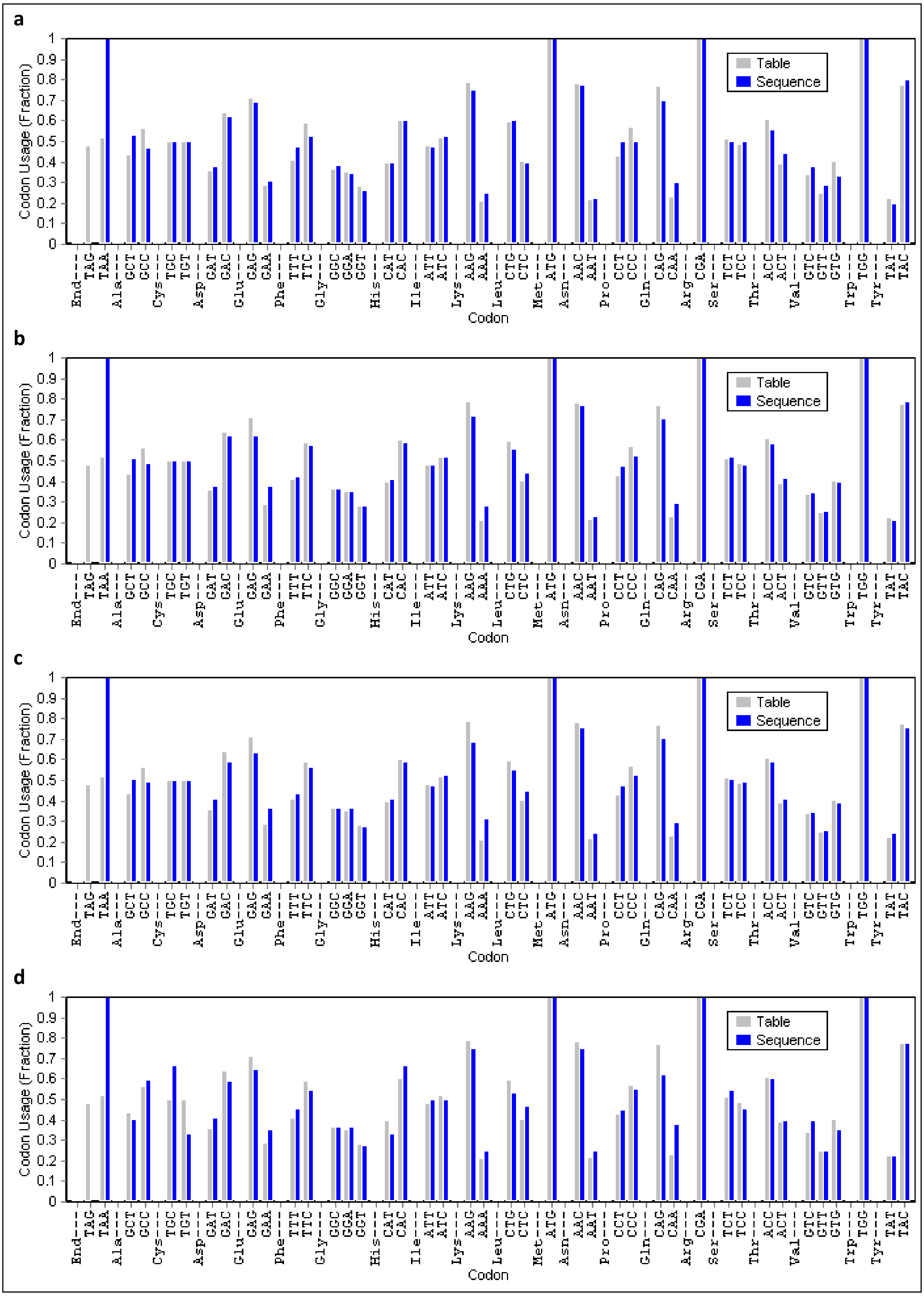

Supplementary Figure 1: Adaptation of synonymous codon usage frequencies of the artificial *pfa* gene cluster C1_V1. Unused codons with frequencies below the applied frequency threshold of 0.2 are not shown in the graph. (a) *pfa1*. (b) *pfa2*. (c) *pfa3*. (d) *ppt*.

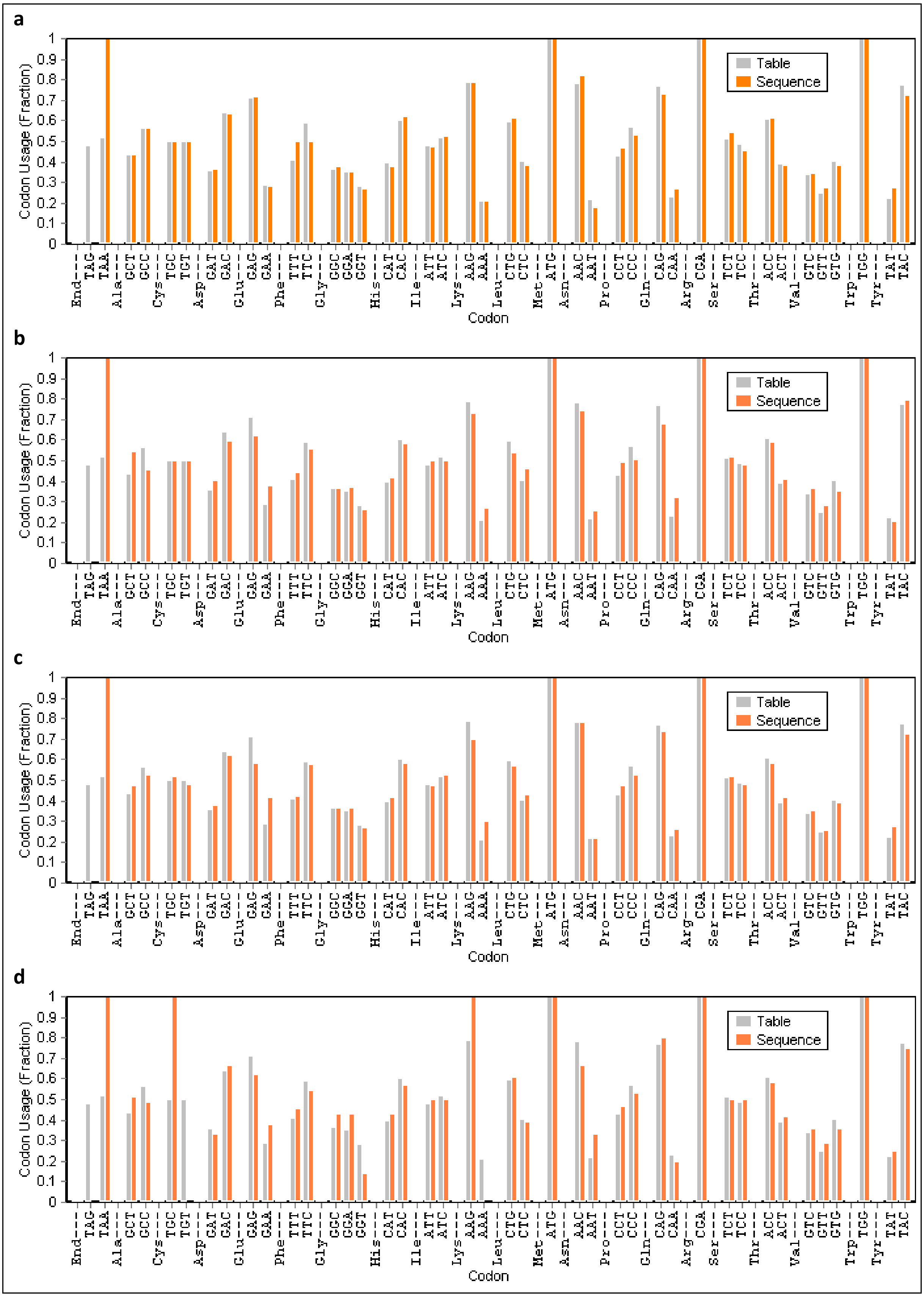

Supplementary Figure 2: Adaptation of synonymous codon usage frequencies of the artificial *pfa* gene cluster C3. Unused codons with frequencies below the applied frequency threshold of 0.2 are not shown in the graph. (a) *pfa1*. (b) *pfa2*. (c) *pfa3*. (d) *ppt*.

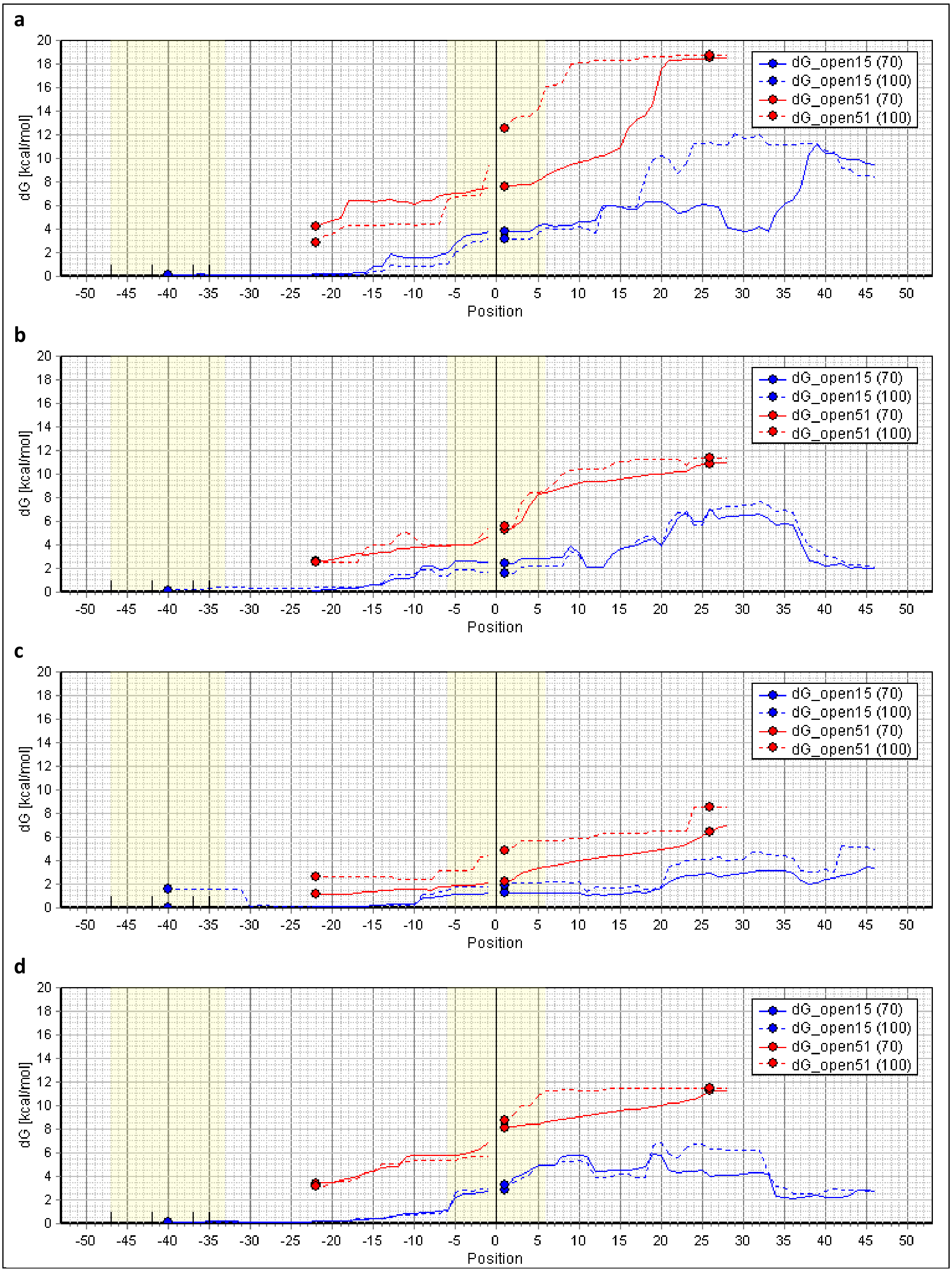

Supplementary Figure 3: Opening energies for the translation initiation sites of cluster C1_V1. Opening energies to make a stretch of 15 nt and 51 nt accessible are drawn as blue and red lines, respectively. Solid and dashed lines correspond to a maximal allowed base pair span of 70 nt and 100 nt, respectively. Position denotes the center of the stretch. The yellow regions correspond to the first stretch at the cap from -47 to -33 (Cap_{15}) and the Kozak sequence. Alternative TSSs are shown as vertical bars[41]. Control points are drawn at -40 (Cap_{15}), the first 51 nt-stretch at -22 (UTR_{51}), the ATG at +1 (ATG_{15}, ATG_{51}), and the center of the last 51 nt-stretch at +26 (CDS_{51}). a) *pfa1*. b) *pfa2*. c) *pfa3*. d) *ppt*.

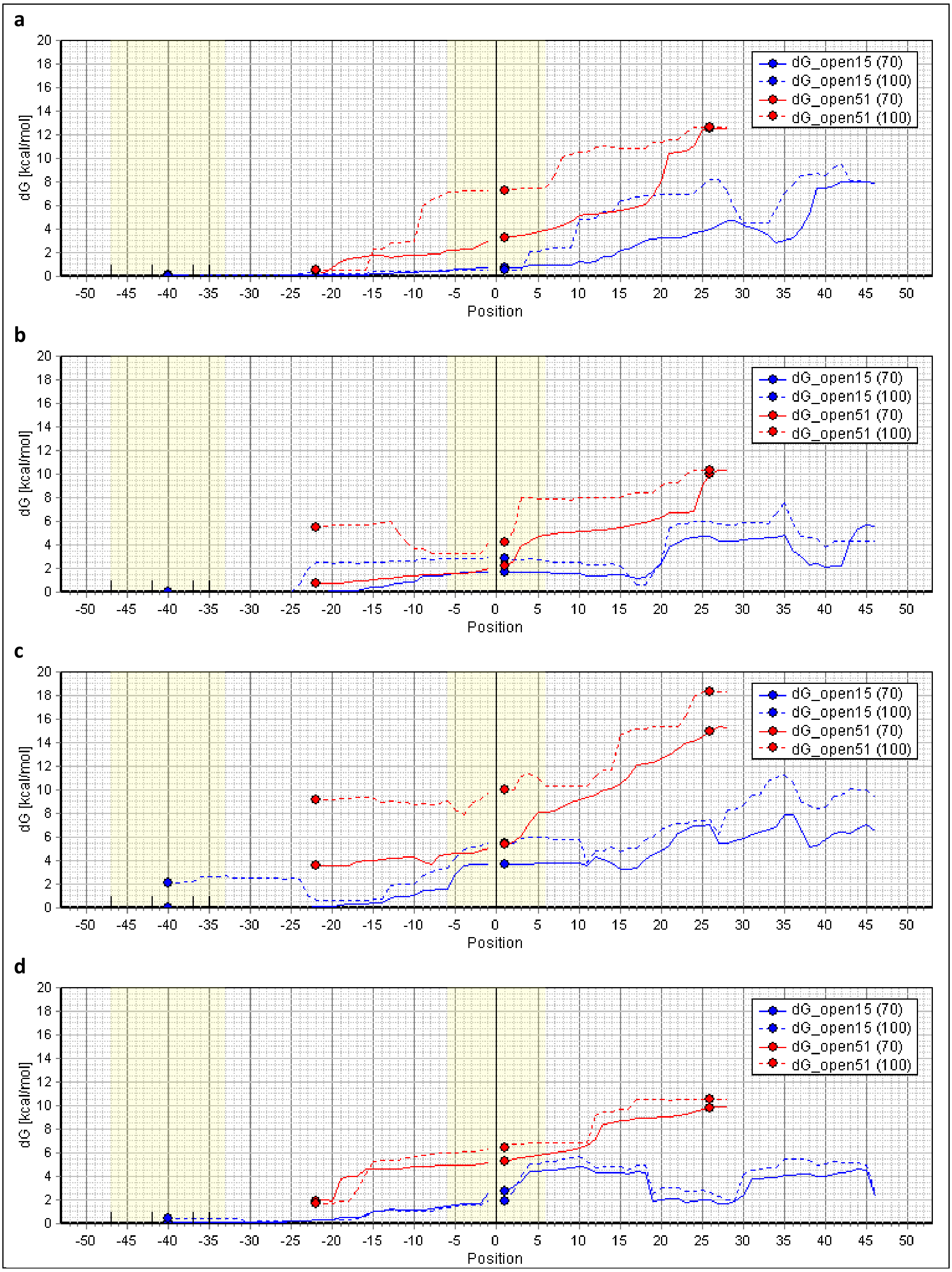

Supplementary Figure 4: Opening energies for the translation initiation sites of cluster C3. Opening energies to make a stretch of 15 nt and 51 nt accessible are drawn as blue and red lines, respectively. Solid and dashed lines correspond to a maximal allowed base pair span of 70 nt and 100 nt, respectively. Position denotes the center of the stretch. The yellow regions correspond to the first stretch at the cap from -47 to -33 (Cap_{15}) and the Kozak sequence. Alternative TSSs are shown as vertical bars[41]. Control points are drawn at -40 (Cap_{15}), the first 51 nt-stretch at -22 (UTR_{51}), the ATG at +1 (ATG_{15}, ATG_{51}), and the center of the last 51 nt-stretch at +26 (CDS_{51}). a) *pfa1*. b) *pfa2*. c) *pfa3*. d) *ppt*.

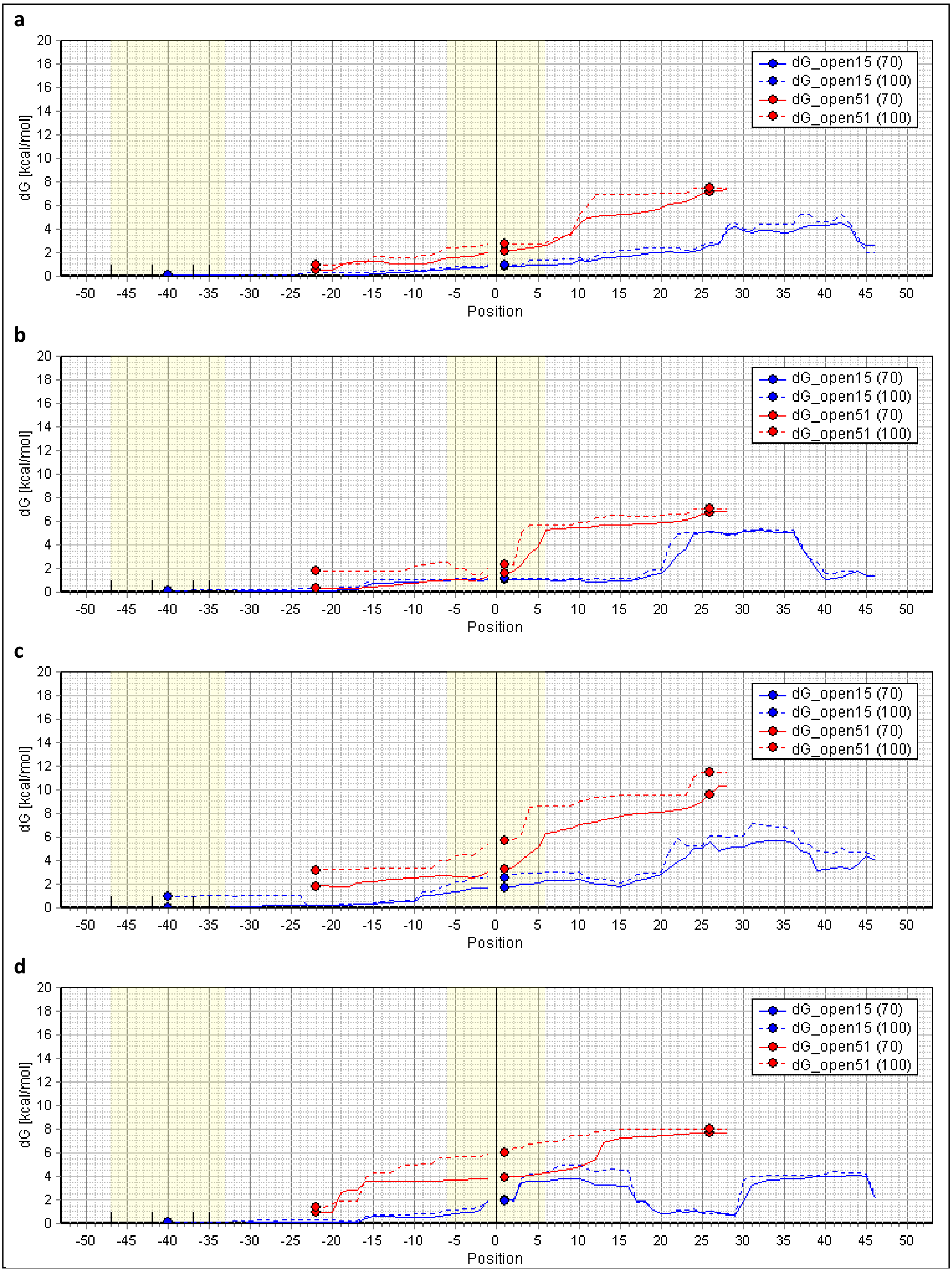

Supplementary Figure 5: Opening energies for the translation initiation sites of cluster C3_mod 5'. Opening energies to make a stretch of 15 nt and 51 nt accessible are drawn as blue and red lines, respectively. Solid and dashed lines correspond to a maximal allowed base pair span of 70 nt and 100 nt, respectively. Position denotes the center of the stretch. The yellow regions correspond to the first stretch at the cap from -47 to -33 (Cap_{15}) and the Kozak sequence. Alternative TSSs are shown as vertical bars[41]. Control points are drawn at -40 (Cap_{15}), the first 51 nt-stretch at -22 (UTR_{51}), the ATG at +1 (ATG_{15}, ATG_{51}), and the center of the last 51 nt-stretch at +26 (CDS_{51}). For details see Supplementary Table 2. a) *pfa1*. b) *pfa2*. c) *pfa3*. d) *ppt*.

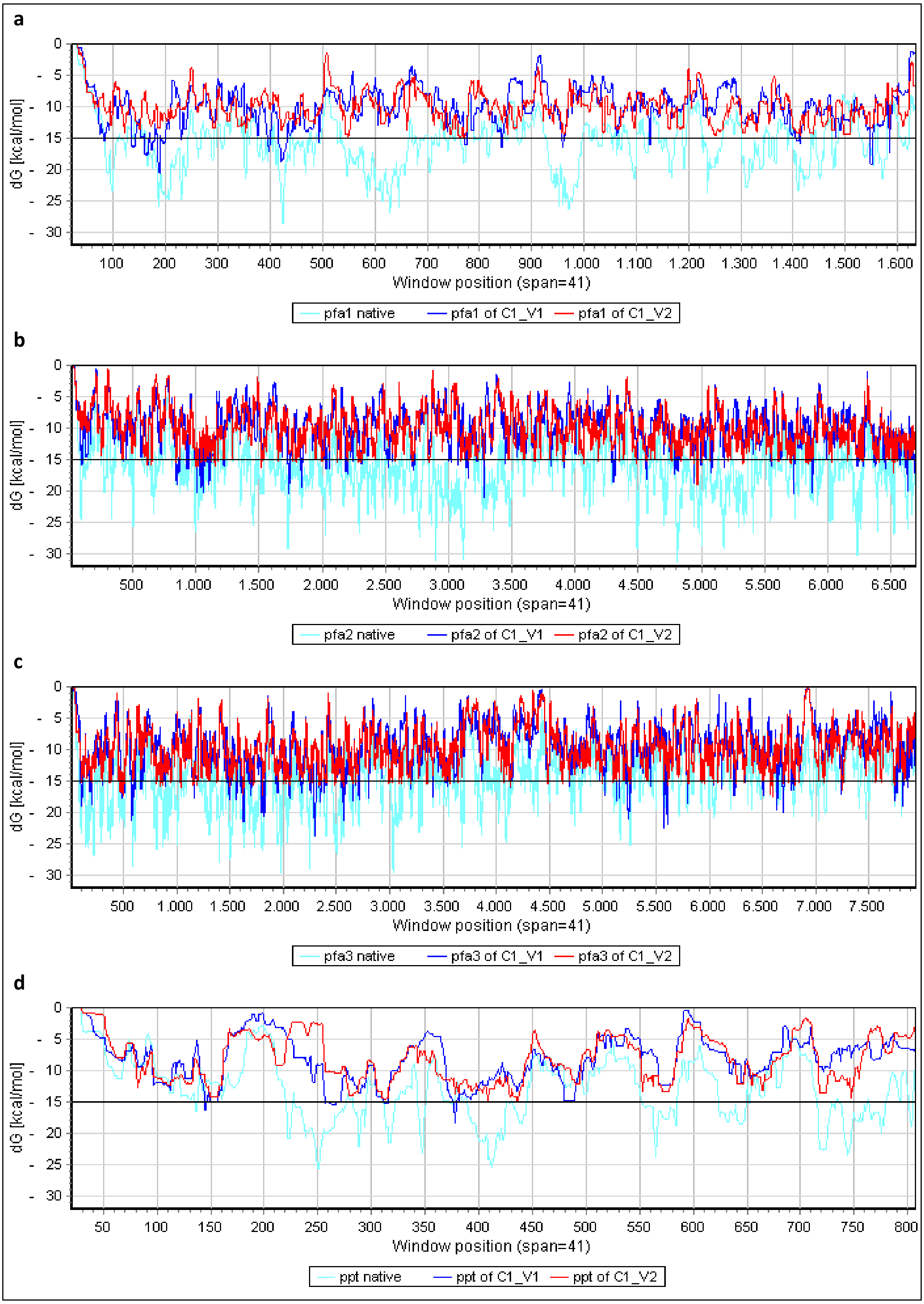

Supplementary Figure 6: Local ensemble free energies calculated from a window of 41 nt of the mRNAs of genes *pfa1*, *pfa2*, *pfa3*, and *ppt* from the native *pfa* biosynthetic gene cluster and from the artificial *pfa* biosynthetic gene clusters C1_V1 and C1_V2. About 95 % of all 41 nt-windows in the genome of *Yarrowia lipolytica* show values above a threshold of -15 kcal/mol. The positions are centered at the windows. a) *pfa1*. b) *pfa2*. c) *pfa3*. d) *ppt*.

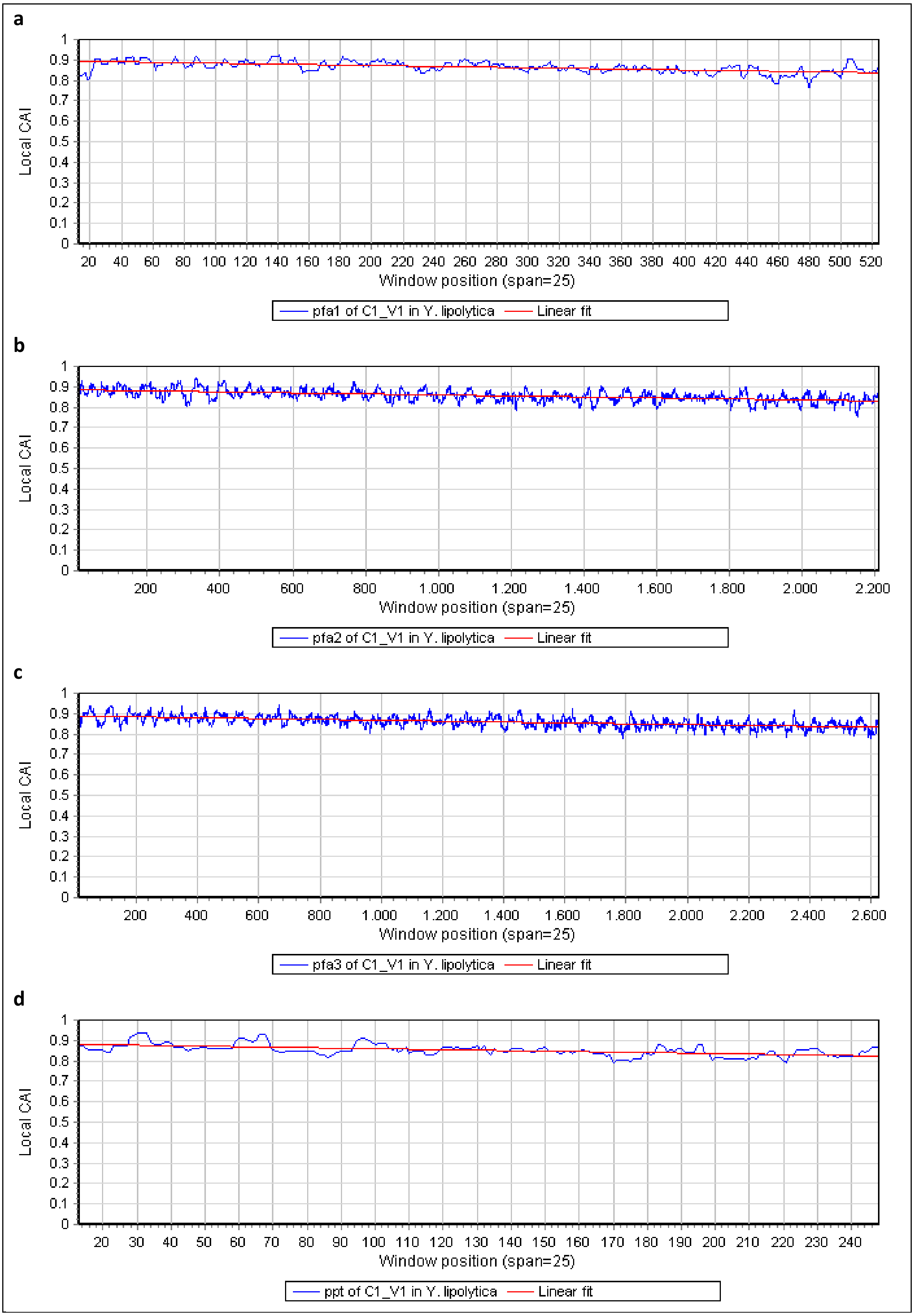

Supplementary Figure 7: Course of the local codon adaptation index (CAI) of the coding sequences of genes *pfa1*, *pfa2*, *pfa3*, and *ppt* from the artificial *pfa* biosynthetic gene clusters C1_V1. Window width is 25 codons. The gradient between start and stop codon of each coding sequence was adjusted to 0.05. The genomic codon table was used for formal CAI calculation. a) *pfa1*. b) *pfa2*. c) *pfa3*. d) *ppt*.

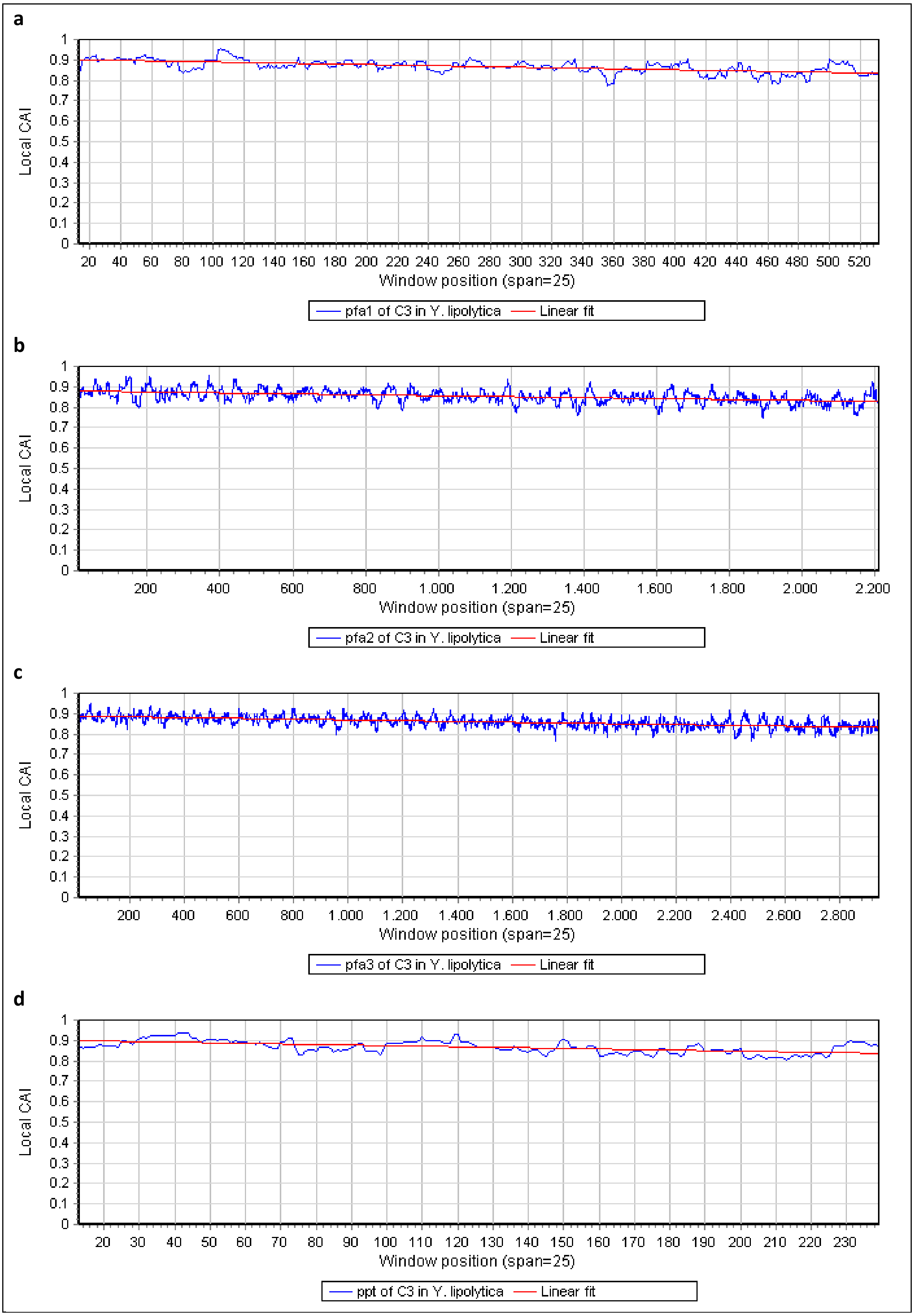

Supplementary Figure 8: Course of the local codon adaptation index (CAI) of the coding sequences of genes *pfa1*, *pfa2*, *pfa3*, and *ppt* from the artificial *pfa* biosynthetic gene clusters C3. Window width is 25 codons. The gradient between start and stop codon of each coding sequence was adjusted to 0.05. The genomic codon table was used for formal CAI calculation. a) *pfa1*. b) *pfa2*. c) *pfa3*. d) *ppt*.

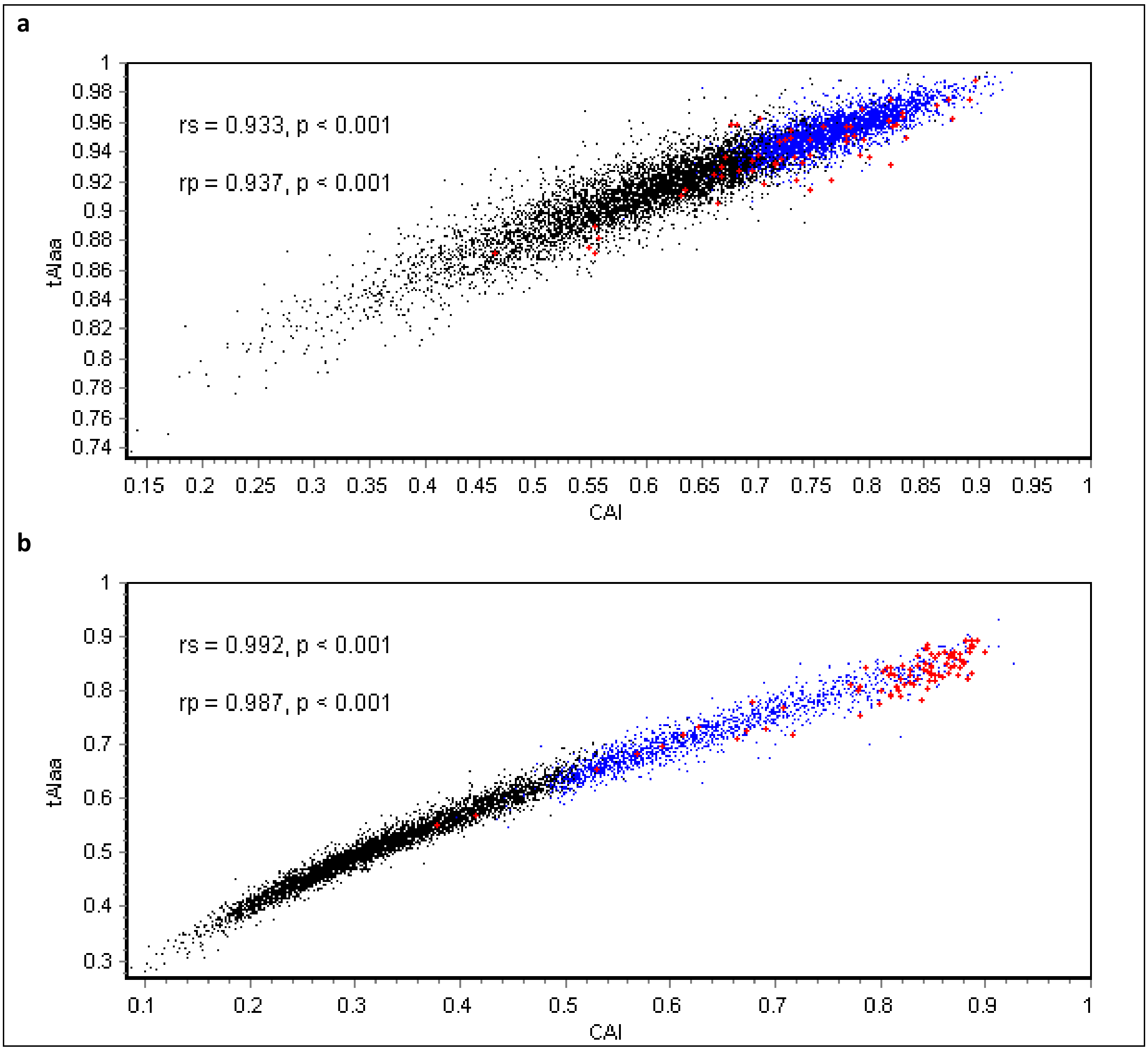

Supplementary Figure 9: Relationship between CAI and tAIaa. a) *Aetherobacter fasciculatus* (SBSr002). b) *Yarrowia lipolytica*. CAI/tAIaa values for every CDS in the genome are represented by black dots. Data points for the automatically generated control set are represented by blue dots and the data points for the reference sets are shown in red. The blue cluster, which in the case of *Y. lipolytica* consists of the set of genes that were used to build the codon table for the optimization of the synonymous codon fractions of version C1_V2, is always located in the upper half of the scatter plot. The reference set of, in the case of *Y. lipolytica* predominantly and in the case of *A. fasciculatus* (SBSr002) exclusively, ribosomal genes, is clearly separated from the bulk for the *Y. lipolytica* sequences. *A. fasciculatus* (SBSr002) shows a more blurred picture in this respect. To avoid an amino acid dependency, a normalized variant of the tAI – tAIaa[20,32,33] (weights scaled to the highest weight within each codon group representing a certain amino acid) - was used. Additionally, analogous to the CAI, Met and Trp are omitted from the calculation. The Percudani-rule[35] for eukaryotes was used for *Y. lipolytica* to determine possible codon-anticodon matchings. Inosine modification for the $base_{34}$ of the anticodon for C-ending codons of Ala, Ile, Leu, Pro, Arg, Ser, Thr, and Val are assumed. A G:U-wobble is used by Cys, Asp, Phe, His, Asn, and Tyr. Additionally, Gly needs a G:U- and a U:G-wobble. Because of uncertainty about tRNA base modifications in the *A. fasciulatus* tRNAnome, also only mandatory wobble interactions were taken into account. Only G:U-wobble interactions are needed, besides the common I:C interaction for Arg. Specificities for watson-crick near I:C interactions were set to 1, all other codon-anticodon interactions using a wobble for the sake of comparability to 0.5. For *Y. lipolytica* 94 % of the codons with the highest adaptiveness scores per synonymous codon group have also the highest tAIaa-weights per group. The corresponding value for *A. fasciculatus* (SBSr002) is 78 %. An analogous examination for the lowest scores yields 89 % and 67 %, respectively[36]. Remark: the choice of much smaller weights stretches the tAIaa-range of the plot for *A. fasciculatus* (SBSr002).

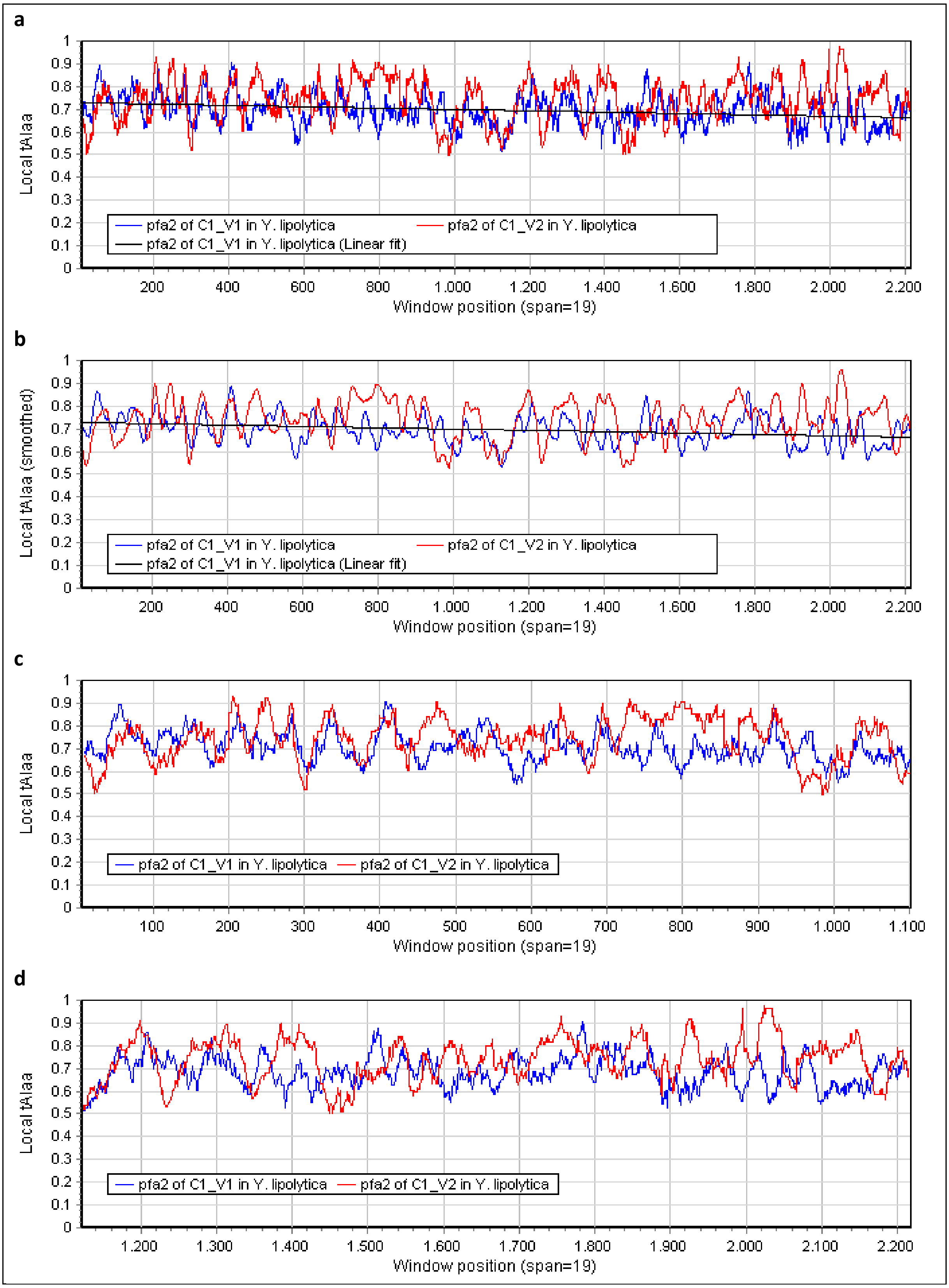

Supplementary Figure 10: Comparison of *pfa2* from the artificial *pfa* biosynthetic gene clusters C1_V1 and C1_V2 based on tRNA adaptation to *Yarrowia lipolytica* CLIB122. (a) *pfa2*. (b) *pfa2* smoothed. (c) 5'-half of *pfa2*. (d) 3'-half of *pfa2*. For the C1_V1 coding sequence of *pfa2* (blue lines) a linear fit is added in (a) and (b), respectively, to show the reproduction of the initially envisioned gradient. The course of the local tAIaa of version C1_V2 is shown in red. In (c) and (d) a detail view of the first and second half of the CDS is shown.

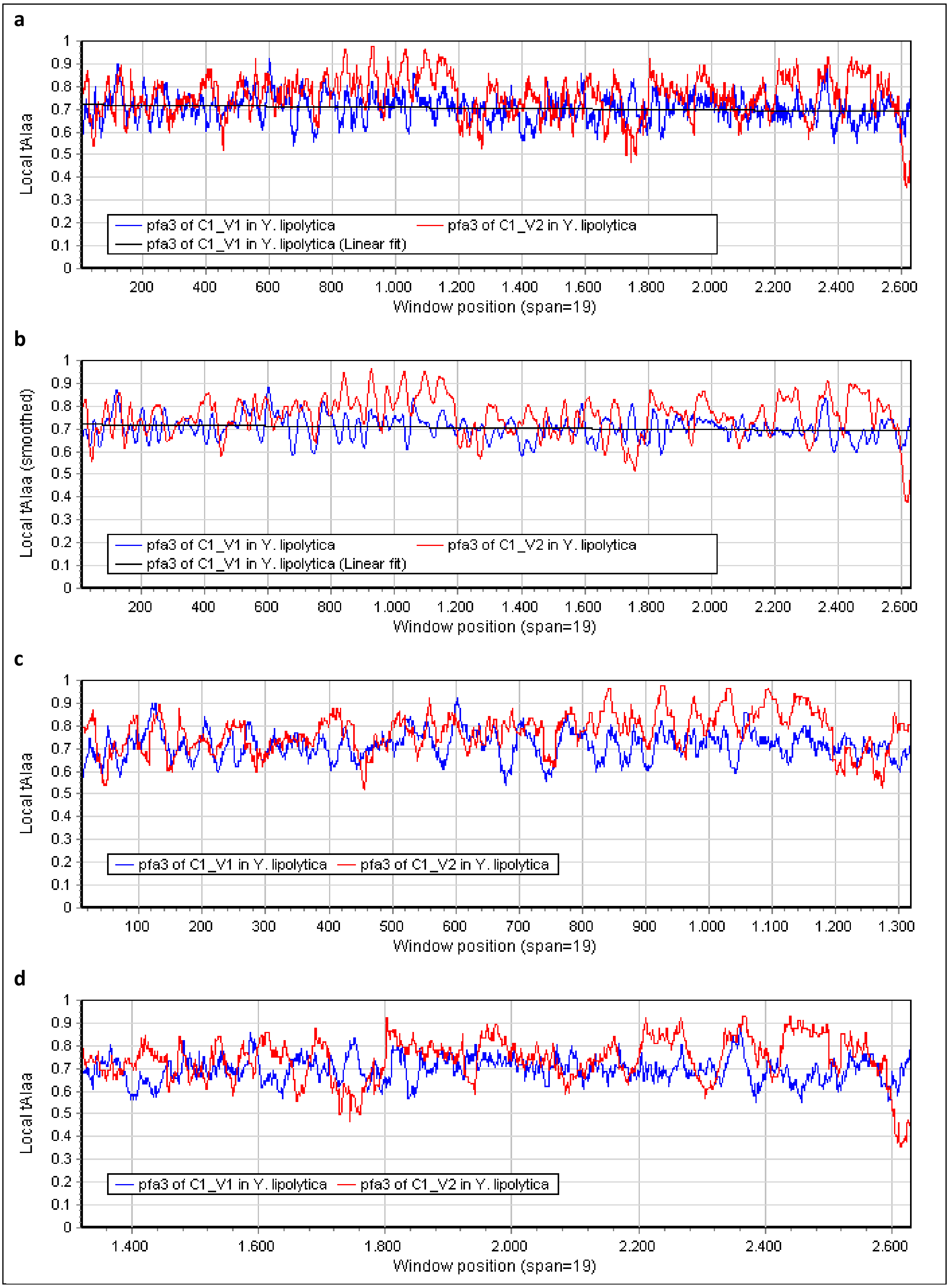

Supplementary Figure 11: Comparison of *pfa3* from the artificial *pfa* biosynthetic gene clusters C1_V1 and C1_V2 based on tRNA adaptation to *Yarrowia lipolytica* CLIB122. (a) *pfa3*. (b) *pfa3* smoothed. (c) 5'-half of *pfa3*. (d) 3'-half of *pfa3*. For the C1_V1 coding sequence of *pfa3* (blue lines) a linear fit is added in (a) and (b), respectively, to show the reproduction of the initially envisioned gradient. The course of the local tAIaa of version C1_V2 is shown in red. In (c) and (d) a detail view of the first and second half of the CDS is shown.

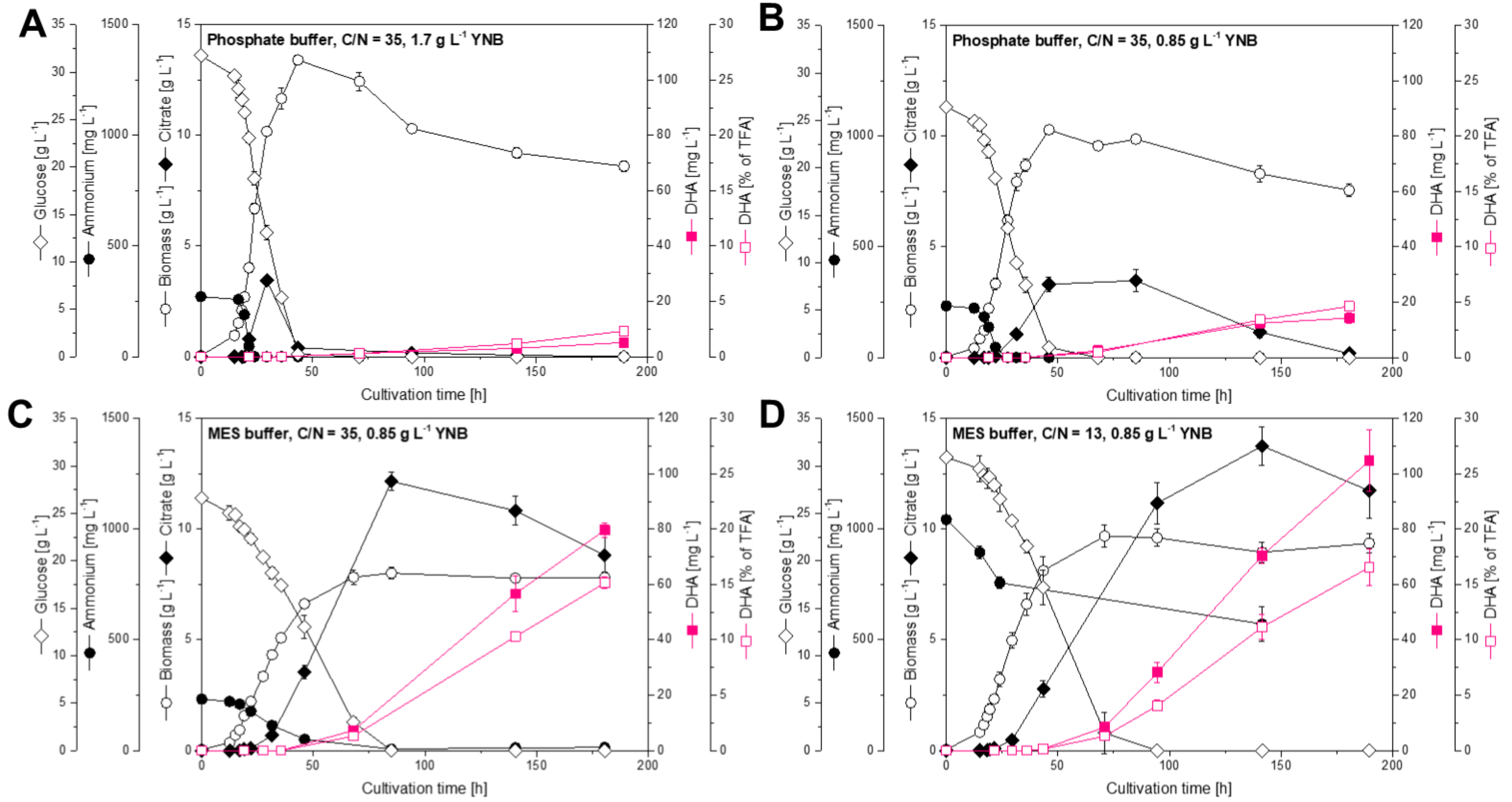

Supplementary Figure 12: DHA production improvement in *Y. lipolytica* Po1h::Af4 based on an optimization of medium composition. Shake flask cultivations were carried out in triplicates (n = 3). Source data are provided as a Source Data file.

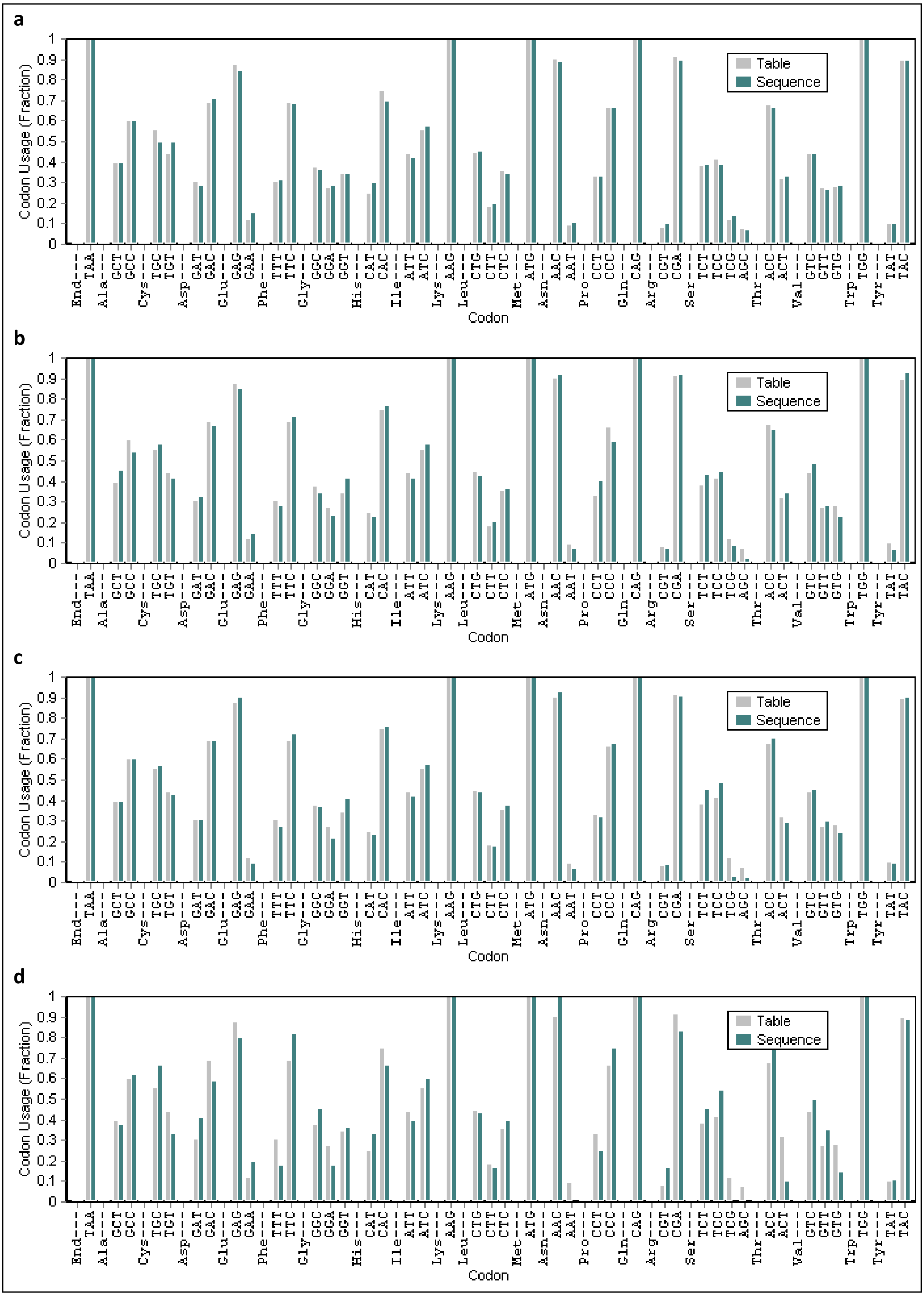

Supplementary Figure 13: Adaptation of synonymous codon usage frequencies of the artificial *pfa* gene cluster C1_V2. The 20 sense codons with the lowest relative adaptiveness values according to Supplementary Table 14 are omitted from the graph. (a) *pfa1*. (b) *pfa2*. (c) *pfa3*. (d) *ppt*.

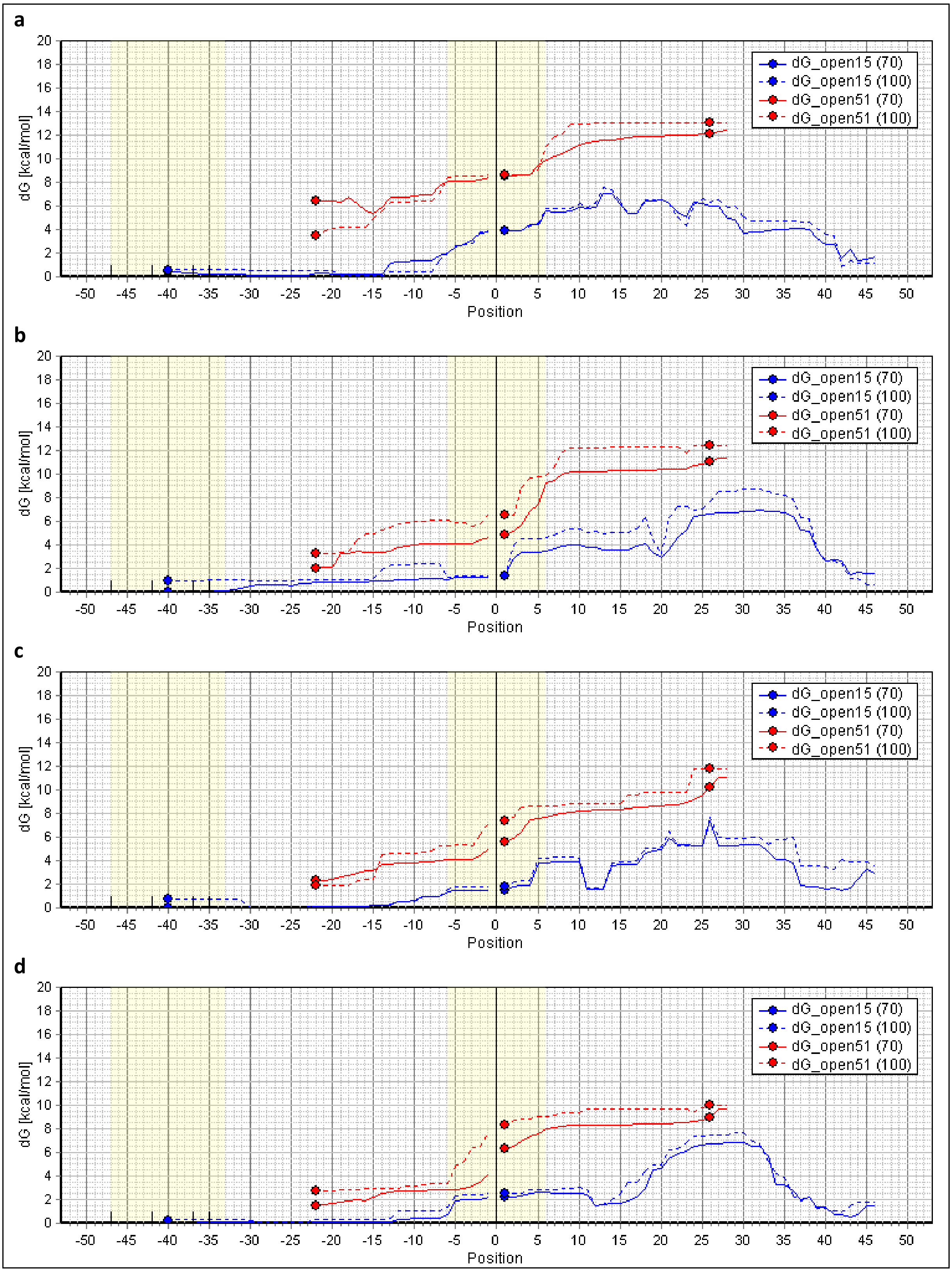

Supplementary Figure 14: Opening energies for the translation initiation sites of cluster C1_V2. Opening energies to make a stretch of 15 nt and 51 nt accessible are drawn as blue and red lines, respectively. Solid and dashed lines correspond to a maximal allowed base pair span of 70 nt and 100 nt, respectively. Position denotes the center of the stretch. The yellow regions correspond to the first stretch at the cap from -47 to -33 (Cap_{15}) and the Kozak sequence. Alternative TSSs are shown as vertical bars[41]. Control points are drawn at -40 (Cap_{15}), the first 51 nt-stretch at -22 (UTR_{51}), the ATG at +1 (ATG_{15}, ATG_{51}), and the center of the last 51 nt-stretch at +26 (CDS_{51}). a) *pfa1*. b) *pfa2*. c) *pfa3*. d) *ppt*.

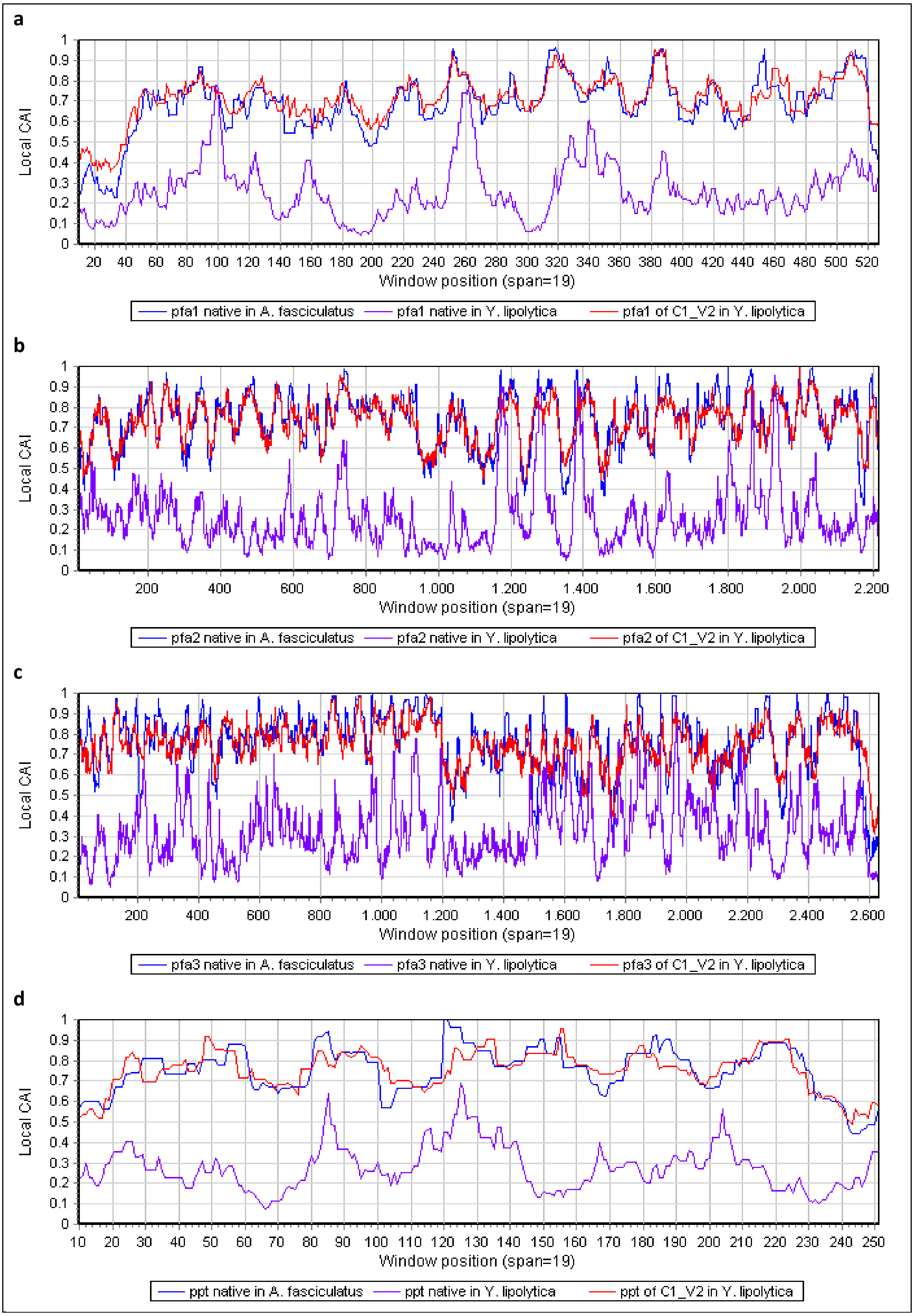

Supplementary Figure 15: Course of the local codon adaptation index (CAI) of the coding sequences from the native *pfa* biosynthetic gene cluster in *Aetherobacter fasciculatus* (SBSr002) (blue), from the artificial *pfa* biosynthetic gene cluster C1_V2 in *Yarrowia lipolytica* (red) and from the native *pfa* biosynthetic gene cluster in *Y. lipolytica* (violet). Window width is 19 codons. a) *pfa1*. b) *pfa2*. c) *pfa3*. d) *ppt*.

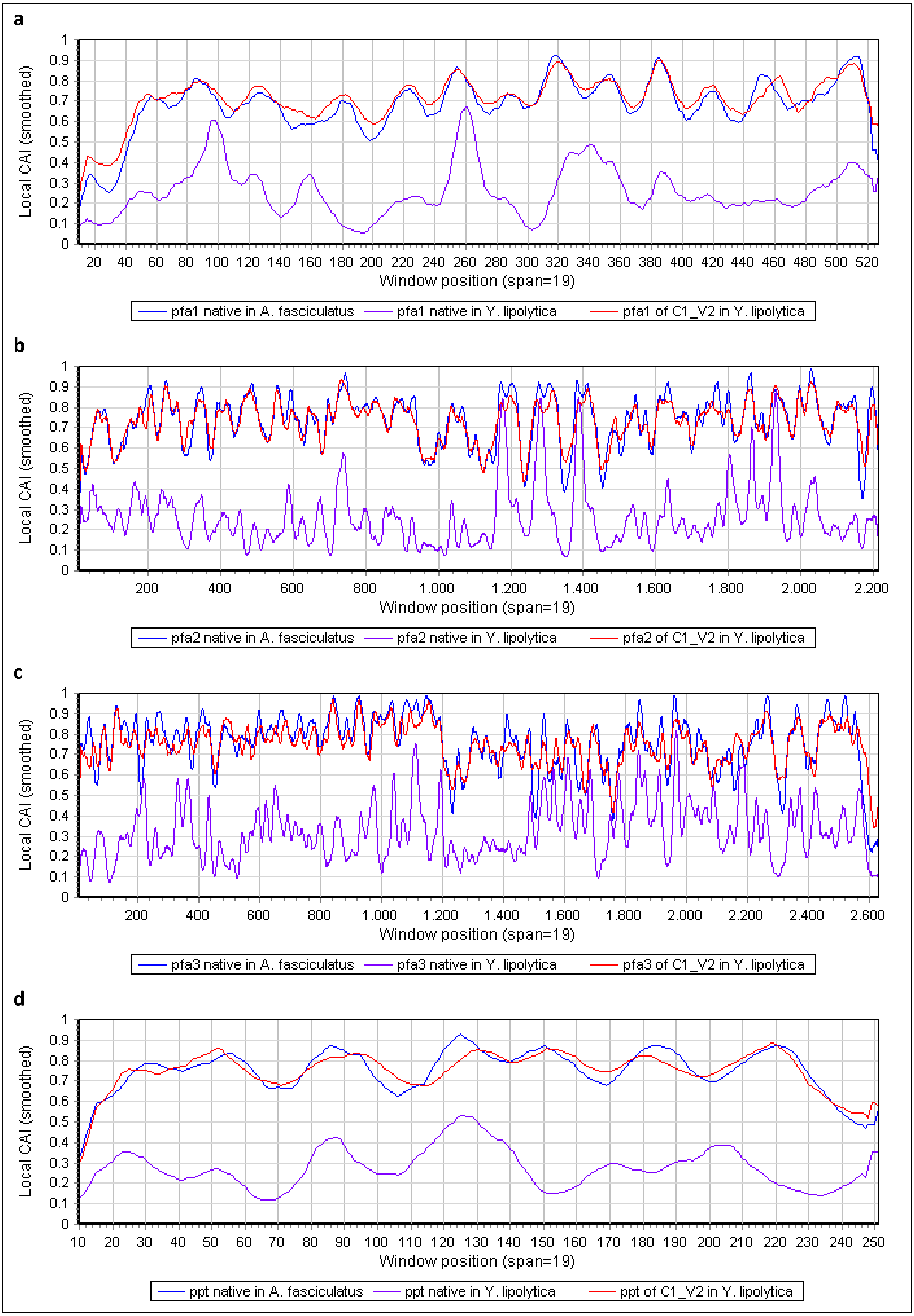

Supplementary Figure 16: Smoothed course of the local codon adaptation index (CAI) of the coding sequences from the native *pfa* biosynthetic gene cluster in *Aetherobacter fasciculatus* (SBSr002) (blue), from the artificial *pfa* biosynthetic gene cluster C1_V2 in *Yarrowia lipolytica* (red) and from the native *pfa* biosynthetic gene cluster in *Y. lipolytica* (violet). Window width is 19 codons. a) *pfa1*. b) *pfa2*. c) *pfa3*. d) *ppt*.

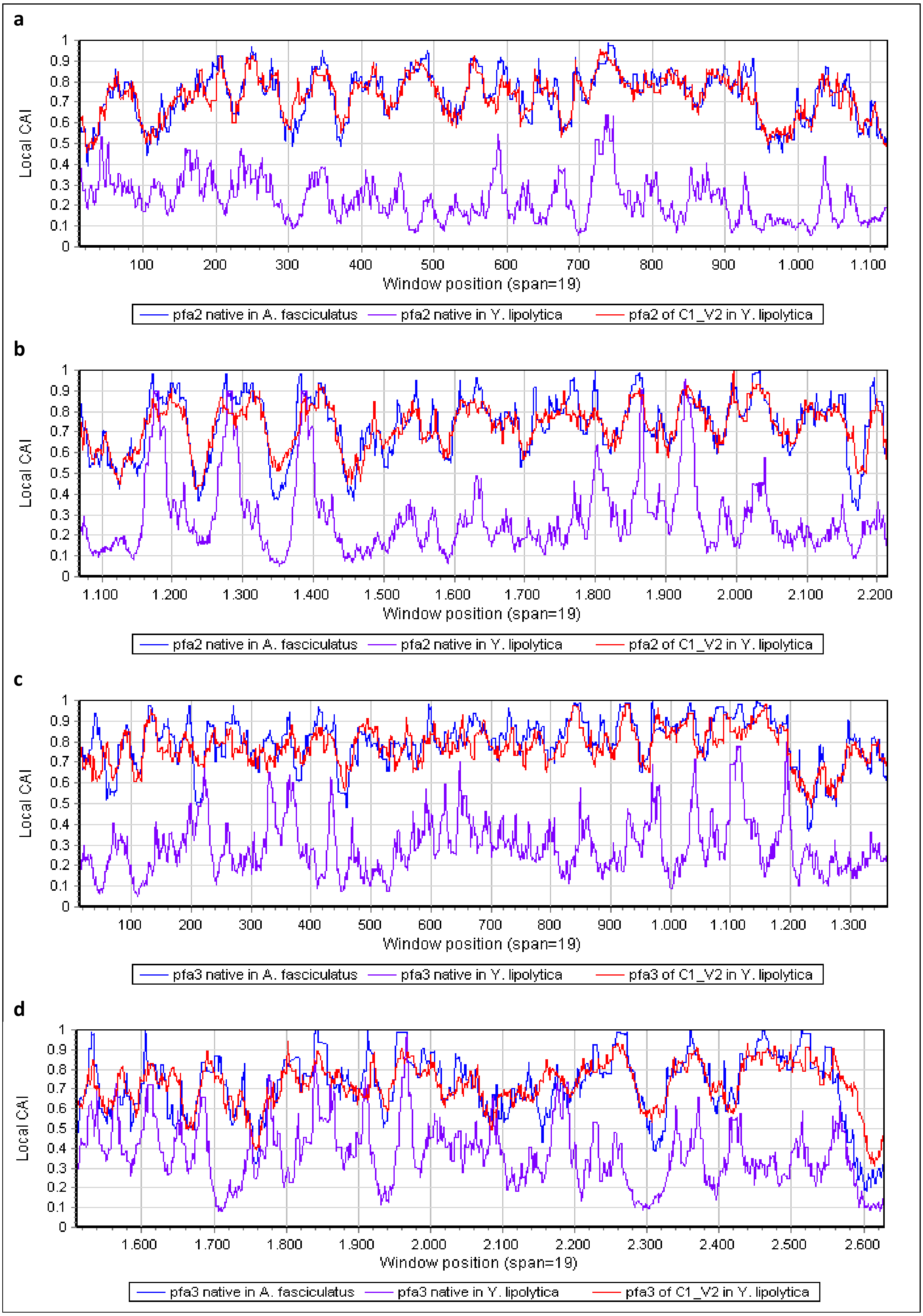

Supplementary Figure 17: Detailed course of the local codon adaptation index (CAI) of genes *pfa2* and *pfa3* from the native *pfa* biosynthetic gene cluster in *Aetherobacter fasciculatus* (SBSr002) (blue), from the artificial *pfa* biosynthetic gene cluster C1_V2 in *Yarrowia lipolytica* (red) and from the native *pfa* biosynthetic gene cluster in *Y. lipolytica* (violet) in detail. Window width is 19 codons. a) *pfa2_5'*. b) *pfa2_3'*. c) *pfa3_5'*. d) *pfa3_3'*.

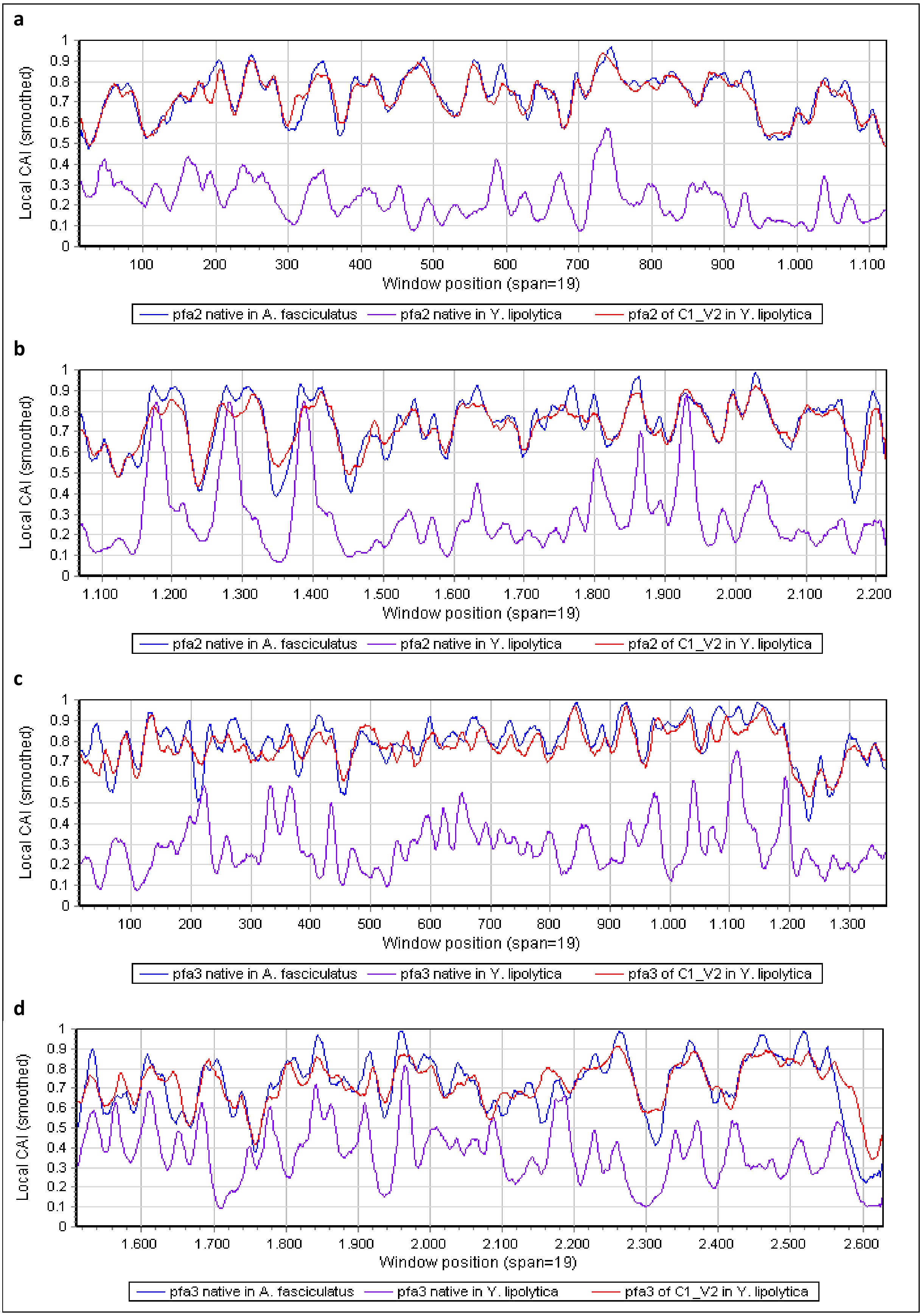

Supplementary Figure 18: Detailed and smoothed course of the local codon adaptation index (CAI) of genes *pfa2* and *pfa3* from the native *pfa* biosynthetic gene cluster in *Aetherobacter fasciculatus* (SBSr002) (blue), from the artificial *pfa* gene cluster C1_V2 in *Yarrowia lipolytica* (red) and from the native *pfa* biosynthetic gene cluster in *Y. lipolytica* (violet) in detail. Window width is 19 codons. A) *pfa2_5'*. B) *pfa2_3'*. C) *pfa3_5'*. D) *pfa3_3'*.

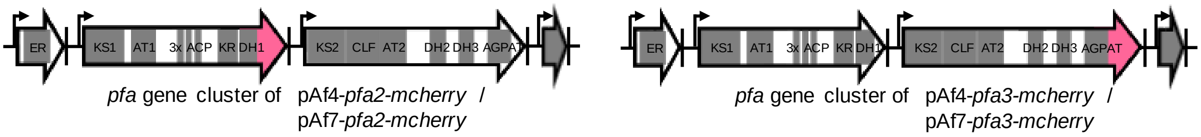

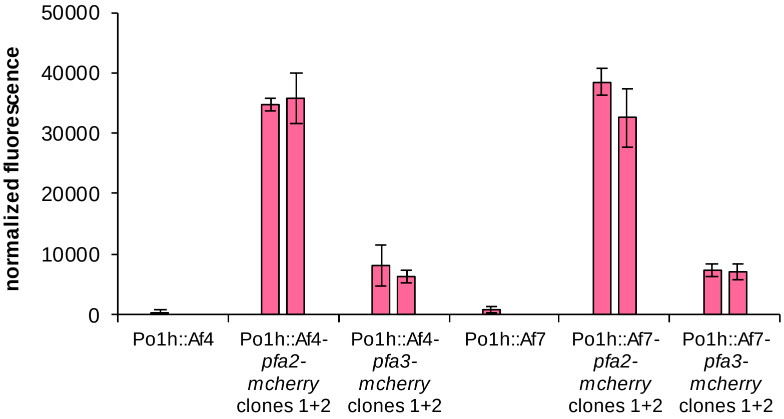

Supplementary Figure 19: mCherry fluorescence signals received from *Yarrowia lipolytica* Po1h::Af4, *Y. lipolytica* Po1h::Af4-*pfa2-mcherry*, *Y. lipolytica* Po1h::Af4-*pfa3-mcherry*, *Y. lipolytica* Po1h::Af7, *Y. lipolytica* Po1h::Af7-*pfa2-mcherry*, and *Y. lipolytica* Po1h::Af7-*pfa3-mcherry*. Cultivations were carried out in 10 mL YNBG medium + 50 mM potassium phosphate buffer pH 6.8 at 28 °C and 200 rpm for 168 h. The indicated values are means and standard deviations of three biological replicates. Source data are provided as a Source Data file.

Supplementary Tables

Supplementary Table 1: Modified and subsequently normalized codon usage table of *Yarrowia lipolytica* CLIB122 generated for the design of the artificial *pfa* gene clusters C1_V1 and C3. Codon counts (n) were obtained from the 6448 protein coding sequences without any length restrictions or offsets. Applying a 20 % threshold to the synonymous codon fractions (f) excluded 23 codons (shaded) and resulted in a subset of 39 sense codons. The synonymous codon fractions were recalculated without the excluded codons (fn). Relative adaptiveness values (w) obtained directly from the given codon counts were used for CAI calculations[4]. Orange and blue marks depict codons that are maximally enriched and depleted, respectively, in a subset of predominantly ribosomal genes.

AA	Cod.	n	f	fn	w	AA	Cod.	n	f	fn	w
A (Ala)	GCC	102886	0.42	0.56	1	P (Pro)	CCC	73374	0.43	0.57	1
	GCT	80207	0.33	0.44	0.78		CCT	54675	0.32	0.43	0.745
	GCA	35003	0.14	0	0.34		CCA	21406	0.13	0	0.292
	GCG	27809	0.11	0	0.27		CCG	21193	0.12	0	0.289
C (Cys)	TGC	19135	0.5	0.5	1	Q (Gln)	CAG	101203	0.77	0.77	1
	TGT	18927	0.5	0.5	0.989		CAA	30504	0.23	0.23	0.301
D (Asp)	GAC	120546	0.64	0.64	1	R (Arg)	CGA	68655	0.43	1	1
	GAT	67689	0.36	0.36	0.562		AGA	26131	0.16	0	0.381
E (Glu)	GAG	145918	0.71	0.71	1		CGG	24146	0.15	0	0.352
	GAA	58861	0.29	0.29	0.403		CGT	18905	0.12	0	0.275
F (Phe)	TTC	72145	0.59	0.59	1		CGC	13697	0.09	0	0.2
	TTT	49765	0.41	0.41	0.69		AGG	7530	0.05	0	0.11
G (Gly)	GGC	68568	0.34	0.37	1	S (Ser)	TCT	68721	0.27	0.51	1
	GGA	65478	0.33	0.35	0.955		TCC	64991	0.25	0.49	0.946
	GGT	52656	0.26	0.28	0.768		TCG	48366	0.19	0	0.704
	GGG	13530	0.07	0	0.197		AGC	30946	0.12	0	0.45
H (His)	CAC	45371	0.6	0.6	1		TCA	24354	0.09	0	0.354
	CAT	29923	0.4	0.4	0.66		AGT	21294	0.08	0	0.31
I (Ile)	ATC	76798	0.5	0.52	1	T (Thr)	ACC	80954	0.42	0.61	1
	ATT	70891	0.46	0.48	0.923		ACT	51514	0.27	0.39	0.636
	ATA	6669	0.04	0	0.087		ACA	33004	0.17	0	0.408
K (Lys)	AAG	146319	0.79	0.79	1		ACG	26632	0.14	0	0.329
	AAA	38829	0.21	0.21	0.265	V (Val)	GTG	80750	0.38	0.41	1
L (Leu)	CTG	105552	0.39	0.6	1		GTC	67973	0.32	0.34	0.842
	CTC	71148	0.26	0.4	0.674		GTT	49958	0.24	0.25	0.619
	CTT	41622	0.15	0	0.394		GTA	12560	0.06	0	0.156
	TTG	32687	0.12	0	0.31	W (Trp)	TGG	37691	1	1	1
	CTA	16533	0.06	0	0.157	Y (Tyr)	TAC	72803	0.77	0.77	1
	TTA	5531	0.02	0	0.052		TAT	21264	0.23	0.23	0.292
M (Met)	ATG	71032	1	1	1	* (Ter)	TAA	2716	0.42	-	-
N (Asn)	AAC	98745	0.78	0.78	1		TAG	2504	0.39	-	-
	AAT	27870	0.22	0.22	0.282		TGA	1228	0.19	-	-

Supplementary Table 2: Local opening energies for various control positions within the translation initiation sites of the three *pfa* genes and gene *ppt* of the artificial *pfa* gene clusters C3 and C3_mod 5'. ΔG values are given in kcal/mol. The calculations related to RNA secondary structures were performed with algorithms from the ViennaRNA Package 2.0[10]. The translation initiation site (TIS) reaches from the first putative transcription start site 47 nt upstream of the ATG start codon down to nucleotide position +53 within the 5' region of the CDS. Using this definition, a region of 100 nt is covered. Five control positions Cap_{15}, UTR_{51}, ATG_{15}, ATG_{51}, and CDS_{51}, were defined to characterize the TIS. The subscripts denote the length of the stretch for which the opening energies were calculated. Positions were fixed according to Supplementary Figs. 4 and 5 (-40, -22, -1, +1, and +26) with the first nucleotide of the start codon at +1. The first control position Cap_{15} (-40) was calculated as the arithmetic mean of the assumed transcription start site positions. In this way, the considered stretch for Cap_{15} covers all putative TSSs. Control position UTR_{51} at -22 is the first possible 51 nt-stretch according to the used definition of the TIS and reaches 4 nt into the 5' region of the CDS. Control position CDS_{51} is the center of the first 51 nt-stretch within the CDS. Calculations were performed for two different maximal allowed base pair spans leading to 10 data points per TIS considered. First with the default settings[10] (maximal base pair span and window size: 70 nt) and second with the same parameters set to 100 nt each. With the latter setting, all bases of the 5' region of the CDS (which were modified by silent mutations) were allowed to engage in bindings with all of the nucleotides of the 5'-UTR. Values that are formally higher for C3_mod 5' than for cluster C3 are underlined.

Gene	ΔG_{open} [kcal/mol]									
	Win=70 / Bps=70					Win=100 / Bps=100				
	Cap_{15}	ATG_{15}	UTR_{51}	ATG_{51}	CDS_{51}	Cap_{15}	ATG_{15}	UTR_{51}	ATG_{51}	CDS_{51}
pfa1 C3	0.1	0.8	0.4	3.3	12.5	0.0	0.5	0.5	7.3	12.6
pfa1 C3_mod 5'	0.1	0.9	0.5	2.1	7.2	0.1	1.0	1.0	2.7	7.5
pfa2 C3	0.0	1.7	0.7	2.2	10.0	0.0	2.9	5.5	4.2	10.4
pfa2 C3_mod 5'	0.0	1.0	0.3	1.5	6.8	0.1	1.1	1.8	2.3	7.0
pfa3 C3	0.0	3.7	3.6	5.4	15.0	2.1	5.4	9.1	10.0	18.3
pfa3 C3_mod 5'	0.0	1.7	1.8	3.2	9.5	0.9	2.6	3.2	5.7	11.5
ppt C3	0.1	2.7	1.9	5.3	9.8	0.4	1.9	1.7	6.4	10.5
ppt C3_mod 5'	0.0	2.0	0.9	3.9	7.7	0.1	1.9	1.4	6.0	8.0

Supplementary Table 3: Percent reduction in local opening energies for various control positions within the translation initiation sites of the three *pfa* genes and gene *ppt* of the artificial *pfa* gene cluster C3_mod 5'. Values up to 1 kcal/mol in the original data are considered too low for comparison. For explanation of the nomenclature see Supplementary Table 2.

Gene	Reduction in ΔG_{open} from C3 to C3_mod 5'									
	Win=70 / Bps=70					Win=100 / Bps=100				
	Cap_{15}	ATG_{15}	UTR_{51}	ATG_{51}	CDS_{51}	Cap_{15}	ATG_{15}	UTR_{51}	ATG_{51}	CDS_{51}
pfa1	-	-	-	-36%	-42%	-	-	-	-63%	-40%
pfa2	-	-41%	-	-32%	-32%	-	-62%	-67%	-45%	-33%
pfa3	-	-54%	-50%	-41%	-37%	-57%	-52%	-65%	-43%	-37%
ppt	-	-26%	-53%	-26%	-21%	-	0%	-18%	-6%	-24%

Supplementary Table 4: Local opening energies for various control positions within the translation initiation sites of the three *pfa* genes and gene *ppt* of the artificial *pfa* gene clusters C1_V1 and C1_V2. ΔG values are given in kcal/mol. For explanation of the nomenclature see Supplementary Table 2.

Gene	ΔG_{open} [kcal/mol]									
	Window=70 / Bps=70					Window=100 / Bps=100				
	Cap_{15}	ATG_{15}	UTR_{51}	ATG_{51}	CDS_{51}	Cap_{15}	ATG_{15}	UTR_{51}	ATG_{51}	CDS_{51}
pfa1 C1_V1	0.1	3.8	4.2	7.6	18.5	0.1	3.2	2.8	12.5	18.7
pfa1 C1_V2	0.4	3.9	6.5	8.5	12.2	0.6	3.9	3.5	8.6	13.1
pfa2 C1_V1	0.0	2.4	2.6	5.2	10.8	0.2	1.6	2.5	5.6	11.3
pfa2 C1_V2	0.0	1.3	2.0	4.9	11.1	1.0	1.4	3.3	6.6	12.4
pfa3 C1_V1	0.0	1.2	1.2	2.2	6.4	1.6	1.8	2.6	4.8	8.5
pfa3 C1_V2	0.0	1.5	2.3	5.6	10.2	0.7	1.8	1.9	7.4	11.8
ppt C1_V1	0.1	2.8	3.4	8.1	11.2	0.1	3.3	3.2	8.7	11.5
ppt C1_V2	0.0	2.2	1.5	6.3	6.3	0.2	2.5	2.7	8.4	10.0

Supplementary Table 5: Average and minimum ensemble free energy (EFE) values calculated from all 41 nt-sized sliding windows of the CDSs of genes *pfa1*, *pfa2*, *pfa3*, and *ppt* from the native *pfa* biosynthetic gene cluster and from the artificial *pfa* biosynthetic gene clusters C1_V1 and C1_V2. ΔG values are given in kcal/mol. The 5'-UTR was not included in the calculations.

Gene	ΔG_{EFE_41} [kcal/mol]	
	ΔG_{mean}	ΔG_{min}
pfa1 native	-15.8	-28.5
pfa1 C1_V1	-10.1	-20.6
pfa1 C1_V2	-10.2	-14.9
pfa2 native	-16.1	-31.3
pfa2 C1_V1	-9.9	-21.1
pfa2 C1_V2	-10.0	-19.0
pfa3 native	-14.3	-29.6
pfa3 C1_V1	-9.6	-23.8
pfa3 C1_V2	-9.7	-17.4
ppt native	-13.3	-25.6
ppt C1_V1	-8.3	-18.3
ppt C1_V2	-8.4	-15.0

Supplementary Table 6: Codon adaptation indices and tAIaa values of the protein coding sequences of genes *pfa1*, *pfa2*, *pfa3*, and *ppt* from the native *pfa* biosynthetic gene cluster and from the artificial biosynthetic gene clusters C1_V1 and C1_V2. CAI calculations are based on the relative adaptiveness weights from Supplementary Table 1 (CAI_G), Supplementary Table 14 (CAI_A), and Supplementary Table 15 (CAI_R). Calculations were performed according to the standard CAI definition[4] omitting Met and Trp. tAIaa calculations are based on the gene copy numbers (GCN) given in Supplementary Table 15.

Gene	CAI_G	CAI_A	CAI_R	tAIaa
pfa1 native	0.628	0.367	0.227	0.429
pfa1 C1_V1	0.862	0.776	0.651	0.706
pfa1 C1_V2	0.867	0.802	0.695	0.721
pfa2 native	0.618	0.357	0.224	0.411
pfa2 C1_V1	0.856	0.762	0.637	0.692
pfa2 C1_V2	0.870	0.815	0.720	0.732
pfa3 native	0.661	0.413	0.291	0.485
pfa3 C1_V1	0.859	0.766	0.640	0.702
pfa3 C1_V2	0.880	0.830	0.740	0.749
ppt native	0.636	0.365	0.259	0.432
ppt C1_V1	0.852	0.760	0.650	0.729
ppt C1_V2	0.860	0.803	0.732	0.74

Supplementary Table 7: Sequence features of the translation initiation site (TIS) and the protein coding sequences (CDS) of genes *pfa1*, *pfa2*, *pfa3*, and *ppt* from the artificial *pfa* biosynthetic gene clusters C1_V1 and C1_V2. TIS features consisting of the arithmetic means of the values for Cap_{15}, UTR_{51}, ATG_{15}, ATG_{51}, and CDS_{51}, calculated for the two different base pair spans from Supplementary Table 4 and CDS features comprising ΔG_{mean} and ΔG_{min} from Supplementary Table 5 as well as CAI_G, CAI_A, CAI_R, and tAIaa from Supplementary Table 6 are given. For explanation of the nomenclature see Supplementary Table 2, 5, and 6.

Gene	TIS - Features					CDS - Features						
	Cap_{15}	ATG_{15}	UTR_{51}	ATG_{51}	CDS_{51}	ΔG_{mean}	ΔG_{min}	CAI_G	CAI_A	CAI_R	tAIaa	$tAIaa_{min}$
pfa1 C1_V1	0.1	3.5	3.5	10.1	18.6	-10.1	-20.6	0.862	0.776	0.651	0.706	0.513
pfa1 C1_V2	0.5	3.9	5.0	8.6	12.6	-10.2	-14.9	0.867	0.802	0.695	0.721	0.426
pfa2 C1_V1	0.1	2.0	2.5	5.4	11.1	-9.9	-21.1	0.856	0.762	0.637	0.692	0.516
pfa2 C1_V2	0.5	1.4	2.6	5.7	11.7	-10.0	-19.0	0.870	0.815	0.720	0.732	0.496
pfa3 C1_V1	0.8	1.5	1.9	3.5	7.4	-9.6	-23.8	0.859	0.766	0.640	0.702	0.540
pfa3 C1_V2	0.3	1.6	2.1	6.5	11	-9.7	-17.4	0.880	0.830	0.740	0.749	0.352
ppt C1_V1	0.1	3.0	3.3	8.4	11.3	-8.3	-18.3	0.852	0.760	0.650	0.729	0.580
ppt C1_V2	0.1	2.3	2.1	7.3	8.1	-8.4	-15.0	0.860	0.803	0.732	0.74	0.460

Supplementary Table 8: Differences in TIS and CDS features between the artificial *pfa* gene clusters C1_V1 and C1_V2. Relative difference is defined as the raw value obtained for C1_V2 subtracted by the raw value obtained for C1_V1 subsequently divided by the arithmetic mean. Lower opening energies are considered better than the higher ones. Therefore, relative difference in quality (concerning overall translation efficiency) for the selected sequence features is shown. Raw opening energies lower than 1 kcal/mol were omitted from the calculation.

Gene	Relative improvement from C1_V1 to C1_V2 [%]											
	TIS - Features					CDS - Features						
	Cap_{15}	ATG_{15}	UTR_{51}	ATG_{51}	CDS_{51}	ΔG_{mean}	ΔG_{min}	CAI_G	CAI_A	CAI_R	tAIaa	$tAIaa_{min}$
pfa1	-	-11%	-35%	16%	38%	-1%	32%	1%	3%	7%	2%	-19%
pfa2	-	35%	-4%	-5%	-5%	-1%	10%	2%	7%	12%	6%	-4%
pfa3	-	-6%	-10%	-60%	-39%	-1%	31%	2%	8%	14%	6%	-42%
ppt	-	26%	44%	14%	33%	-1%	20%	1%	6%	12%	2%	-23%

Supplementary Table 9. LC-PUFAs produced by *Yarrowia lipolytica* Po1h using myxobacterial PUFA synthases. Cultivation was carried out in 10 mL YNBG medium + 50 mM potassium phosphate buffer pH 6.8 at 28 °C and 200 rpm for 168 h. The indicated values are means and (standard deviations) of three biological replicates. EDA, eicosadienoic acid; ETrA, eicosatrienoic acid; AA, arachidonic acid; DTrA, docosatrienoic acid; DTA, docosatetraenoic acid; DPA, docosapentaenoic acid; DHA, docosahexaenoic acid; TTrA, tetracosatrienoic acid; TTA, tetracosatetraenoic acid. Source data are provided as a Source Data file.

LC-PUFA	Relative amount [% of total fatty acids]		Absolute amount [mg g^{-1} cell dry weight]		Yield [mg L^{-1}]	
	**Po1h::Af4**	**Po1h::Af7**	**Po1h::Af4**	**Po1h::Af7**	**Po1h::Af4**	**Po1h::Af7**
***n*-6 DPA (22:5)**	0.3 (0.0)	0.3 (0.0)	0.3 (0.0)	0.3 (0.0)	2.1 (0.2)	2.2 (0.2)
***n*-3 DPA (22:5)**	0.1 (0.0)	0.0 (0.0)	0.1 (0.0)	0.1 (0.0)	0.4 (0.2)	0.4 (0.2)
**DHA (22:6, *n*-3)**	10.5 (1.0)	10.6 (0.3)	12.8 (0.4)	12.2 (0.8)	86.1 (3.6)	88.7 (1.6)
	**Po1h::Mr1**	**Po1h::Mr2**	**Po1h::Mr1**	**Po1h::Mr2**	**Po1h::Mr1**	**Po1h::Mr2**
***n*-7 EDA (20:2)**	0.0 (0.0)	0.0 (0.0)	0.0 (0.0)	0.0 (0.0)	0.1 (0.1)	0.1 (0.0)
***n*-9 ETrA (20:3)**	0.3 (0.0)	0.3 (0.0)	0.3 (0.0)	0.2 (0.0)	1.9 (0.3)	1.8 (0.0)
***n*-7 ETrA (20:3)**	0.4 (0.0)	0.3 (0.0)	0.4 (0.1)	0.3 (0.0)	3.0 (0.4)	2.0 (0.0)
***n*-6 ETrA (20:3)**	0.0 (0.0)	0.0 (0.0)	0.0 (0.0)	0.0 (0.0)	0.0 (0.0)	0.3 (0.3)
***n*-3 ETrA (20:3)**	0.1 (0.0)	0.1 (0.0)	0.1 (0.0)	0.1 (0.0)	0.4 (0.2)	0.5 (0.0)
**AA (20:4, *n*-6)**	1.0 (0.1)	1.7 (0.0)	1.2 (0.1)	1.5 (0.0)	8.0 (0.9)	10.9 (0.5)
***n*-9 DTrA (22:3)**	0.1 (0.0)	0.1 (0.1)	0.1 (0.0)	0.1 (0.1)	0.4 (0.1)	0.4 (0.6)
***n*-7 DTrA (22:3)**	0.5 (0.1)	0.7 (0.0)	0.6 (0.2)	0.6 (0.0)	3.9 (1.1)	4.7 (0.1)
***n*-6 DTA (22:4)**	1.3 (0.1)	1.9 (0.1)	1.4 (0.3)	1.7 (0.1)	9.9 (1.4)	12.5 (0.8)
***n*-7 TTrA (24:3)**	0.1 (0.1)	0.1 (0.1)	0.1 (0.1)	0.1 (0.1)	0.4 (0.4)	0.5 (0.6)
***n*-6 TTA (24:4)**	0.8 (0.1)	1.2 (0.0)	0.9 (0.1)	1.1 (0.0)	6.1 (0.7)	8.1 (0.4)

Supplementary Table 10: DHA production improvement in *Y. lipolytica* Po1h::Af4 based on an optimization of medium composition. Shake flask cultivations were carried out in triplicates (n = 3). Performance parameters were determined after 200 h of cultivation. Source data are provided as a Source Data file.

#	Condition description	**Productivity** [mg_{DHA} L^{-1} h^{-1}]		**Titer** [mg_{DHA} L^{-1}]		**DHA** [% of total FAs]		**$Y_{X/S}$** [g_{DCW} C-mole^{-1}]		**$Y_{P/X}$** [mg_{DHA} g_{DCW}^{-1}]	
0	Glucose (0.666 C-mole L^{-1}, 0.076 N-mole L^{-1}, C/N 8.8, 1.7 g L^{-1} YNB, 50 mM P buffer)	0.51	±0.02	86.10	±3.22	10.54	±0.98	10.12	±0.46	12.78	±0.32
1	Glucose (0.333 C-mole L^{-1}, 0.076 N-mole L^{-1}, C/N 4.4, 1.7 g L^{-1} YNB, 200 mM P buffer)	0.10	±0.00	19.76	±0.79	7.11	±0.36	9.89	±0.35	6.67	±1.51
2	Glucose (0.333 C-mole L^{-1}, 0.076 N-mole L^{-1}, C/N 4.4, 1.7 g L^{-1} YNB, 200 mM MES buffer)	0.16	±0.01	38.69	±1.42	13.54	±0.29	13.06	±0.34	10.50	±0.57
3	Glucose (0.9 C-mole L^{-1}, 0.028 N-mole L^{-1}, C/N 35, 0.85 g L^{-1} YNB, 200 mM MES buffer)	0.44	±0.01	79.73	±2.67	15.15	±0.52	8.81	±0.17	10.20	±0.19
4	Glucose (0.9 C-mole L^{-1}, 0.076 N-mole L^{-1}, C/N 13, 0.85 g L^{-1} YNB, 200 mM MES buffer)	**0.55**	**±0.06**	**104.79**	**±11.23**	**16.56**	**±1.69**	8.69	±0.15	11.21	±1.44
5	Glucose (0.9 C-mole L^{-1}, 0.028 N-mole L^{-1}, C/N 35, 0.85 g L^{-1} YNB, 200 mM P buffer)	0.08	±0.01	14.30	±1.81	4.62	±0.16	10.36	±0.02	1.89	±0.24
6	Glucose (0.9 C-mole L^{-1}, 0.028 N-mole L^{-1}, C/N 35, 1.7 g L^{-1} YNB, 200 mM P buffer)	0.03	±0.00	5.15	±0.52	2.33	±0.13	10.21	±0.07	0.60	±0.07
7	Glycerol (0.333 C-mole L^{-1}, 0.076 N-mole L^{-1}, C/N 4.4, 1.7 g L^{-1} YNB, 200 mM P buffer)	0.24	±0.02	47.89	±4.91	11.77	±2.33	13.25	±1.09	12.80	±2.75
8	Glycerol (0.333 C-mole L^{-1}, 0.076 N-mole L^{-1}, C/N 4.4, 1.7 g L^{-1} YNB, 200 mM MES buffer)	0.30	±0.02	77.48	±5.90	12.11	±0.24	15.65	±0.04	14.55	±0.91
9	Glycerol (0.333 C-mole L^{-1}, 0.076 N-mole L^{-1} (NH_4Cl), C/N 4.4, 1.7 g L^{-1} YNB, 200 mM MES buffer)	0.39	±0.01	75.74	±1.21	13.35	±0.35	14.48	±0.19	14.53	±0.63
10	Glycerol (0.98 C-mole L^{-1}, 0.016 N-mole L^{-1}, C/N 60, 0.85 g L^{-1} YNB, 200 mM MES buffer)	**0.53**	**±0.03**	**112.53**	**±5.92**	10.56	±0.50	5.75	±0.08	**18.45**	**±0.79**
11	Glucose+Glycerol (0.333 C-mole L^{-1}, 0.076 N-mole L^{-1}, C/N 4.4, 1.7 g L^{-1} YNB, 200 mM MES buffer)	0.23	±0.00	58.85	±1.49	14.43	±2.76	14.45	±0.41	13.26	±0.20
12	Acetate (0.6 C-mole L^{-1}, 0.076 N-mole L^{-1}, C/N 8, 1.7 g L^{-1} YNB, 200 mM P buffer)	0.20	±0.00	42.10	±0.71	**16.78**	**±0.72**	6.02	±0.16	**16.39**	**±0.71**

Supplementary Table 11: Primers used for cloning of integrative plasmids in this study. The introduced restriction sites are underlined and the introduced nucleotide exchanges are shown in bold.

Primer	Sequence (5'→3')
HA+*Sda*I+cm^{R} for pINA1312_fwd	*GAGACTGAAATAAATTTAGTCTGCAGCCCAAGCTAGCTTATCGATACGCG*CCTGCAGGATTTAACGACCCTGCCCTGAACCGACG
HA+*Pac*I+cm^{R} for pINA1292+1312_rev	*TACCCTGTCGGATGACTAACTCTCCAGAGCGAGTGTTACACATGGAATTC*TTAATTAAGTAAGTTGGCAGCATCACCCGACGC
cm^{R}_*Sda*I+*Pac*I+*Swa*I+*Sgs*I_fwd	ATGCCTGCAGGTTAATTAATTTAAATGGCGCGCCTGAACCGACGACCGGGTC
p15A_*Sgs*I+*Not*I+*Sgr*DI_rev	GCTCGTCGACGTAGCGGCCGCGGCGCGCCGTATGGGGCTGACTTCAGGTG
*hph*_A_fwd	AATGAAAAAGCCTGAACTCACCGCGACGTC
*hph*_*Bam*HI_rev	GTCCTGGATCCCTATTCCTTTGCCCTCGGACGAG
UAS1B_*Sda*I_fwd	AAGCCTGCAGGTAGCTTATCGATACGCGTGC
hph+*LIP2*t overlap_rev	GGTAGAAGTTGTAAAGAGTGATAAATAGCCTATTCCTTTGCCCTCGGACG
*LIP2*t+*hph* overlap_fwd	CGTCCGAGGGCAAAGGAATAGGCTATTTATCACTCTTTACAACTTCTACC
*LIP2*t_*Sgr*DI_rev	ATCCGTCGACGGTTTCGATTTGTCTTAGAGG
PIS_fragment1_*Not*I_fwd	GCCGCGGCCGCTGGTTACTTTTTGATACACCTG
PIS_fragment1_*Pci*I+*Sgr*DI_rev	AACCGTCGACGACATGTGTCGCAACTTCAAGGAGGACCAAGCTCTGTACACCGAGAAACACCGCTGGAAACGGTGGAAC
PIS_fragment2_*Pac*I_fwd	AGGTTAATTAATTGCGAGTACGACATGGTGG
PIS_fragment2_*Swa*I_rev	GCCATTTAAATTGTTGAGATATACTTCAGACGCTG
MCS for pACYC_fwd	TTTAAACCTGCAGGGCGGTGCACGCGCCATGGCGCGTCGACCGCAACGTTGCGCGACG
MCS for pACYC_rev	GGATCCTTAATTAACGCCTAGGGCGCGCCGGCGCGCGACGTCGCGCAACGTTGCGGTCGACGC
*pfa2*_fwd	CGCTGAGCGACGACGTCGAGG
pfa2+*mcherry* overlap_rev	CCTCCTCGCCCTTGCTCACCATAGAAGCGGGACCGGTCTCCAC
pfa2+*mcherry* overlap_rev_2	CCTCCTCGCCCTTGCTCACCATCGAGGCGGGGCCAGTTTCAACC
mcherry+*pfa2* overlap_fwd	GTGGAGACCGGTCCCGCTTCTATGGTGAGCAAGGGCGAGGAGG
mcherry+*pfa2* overlap_fwd_2	GGTTGAAACTGGCCCCGCCTCGATGGTGAGCAAGGGCGAGGAGG
mcherry+*LIP2*t overlap_rev	GAGGTAGAAGTTGTAAAGAGTGATAAATAGCTCACTTGTACAGCTCGTCCATGCC
*LIP2*t+*mcherry* overlap_fwd	GGCATGGACGAGCTGTACAAGTGAGCTATTTATCACTCTTTACAACTTCTACCTC
linker 2_rev	AACGTTTCGGCGAGAAGCAGGC
*pfa3*_fwd	GAATCTCGGCATGCGAGTCGTGGC
pfa3+*mcherry* overlap_rev	CCTCCTCGCCCTTGCTCACCATACCTCCCTCGACGAGCAGGGTG
pfa3+*mcherry* overlap_rev_2	CCTCCTCGCCCTTGCTCACCATTCCACCCTCAACCAGAAGAGTAGC
mcherry+*pfa3* overlap_fwd	CACCCTGCTCGTCGAGGGAGGTATGGTGAGCAAGGGCGAGGAGG

Continuation of Supplementary Table 11.

Primer	Sequence (5'→3')
mcherry+pfa3 overlap_fwd_2	GCTACTCTTCTGGTTGAGGGTGGAATGGTGAGCAAGGGCGAG GAGG
linker 3_rev	CATGCAACTCCTAGGACAGGTGCC
DH1-Mr_*Kfl*I_fwd	GGGTCCCTGGGAAGGAGGCATG
KR-Mr_fwd	GTCGACGTGGATGAGGCTGTTGC
KR-Mr+DH1-Af overlap_rev	CGAGCTCAATTTCGTCAGCGGATCCAGAGAGCTCG
DH1-Af+KR-Mr overlap_fwd	CGAGCTCTCTGGATCCGCTGACGAAATTGAGCTCG
H2270A_fwd_2	GAGGGTTCTCTGTGGGTGGACGGCAAAC
H2270A_CA to GC_rev	CAGGTGGGTCGA**GC**GTCACCCAGCCAG
H2270A_CA to GC_fwd	CTGGCTGGGTGAC**GC**TCGACCCACCTG
H2270A_rev	GCAACTCCTAGGACAGGTGCCGGTTTCG

Supplementary Table 12: Plasmids constructed in this study. *oriV*, origin of replication; *hph*, hygromycin resistance gene; *cat*, chloramphenicol resistance gene; *aph(3')-IIa*, kanamycin resistance gene; *URA3*, gene encoding the orotidine 5'-phosphate decarboxylase from *Y. lipolytica*; hp4d, hybrid promoter comprising four copies of the distal upstream activating sequence from the *XPR2* promoter in front of a minimal *LEU2* promoter from *Y. lipolytica*; *LIP2*t, terminator of gene *LIP2* encoding an extracellular lipase from *Y. lipolytica*; *XPR2*t, terminator of gene *XPR2* encoding an alkaline extracellular protease from *Y. lipolytica*; zeta, long terminal repeats (LTRs) of the retrotransposon Ylt1 from *Y. lipolytica*.

Plasmid	Characteristics
pINA1312-*Sda*I-cm^R-*Pac*I	Derivative of pINA1312[42] in which hp4d and *XPR2*t were exchanged for *cat* flanked by *Sda*I and *Pac*I restriction sites by Red/ET recombineering. pMB1 *oriV*, *aph(3')-IIa*, *cat*, *URA3*, zeta.
pINA1312-*hph*	Derivative of pINA1312[42] in which *hph* was inserted via *Pml*I and *Bam*HI
pKG1	p15A *oriV* and *cat* of pACYC184 (New England Biolabs) plus hp4d-*hph*-*LIP2*t flanked by *Sgs*I-*Not*I-*Sgr*DI and *Sda*I-*Pac*I-*Swa*I-*Sgs*I restriction sites
pKG1-PIS	Derivative of pKG1 in which 1 kb homology regions upstream and downstream of the preferred integration site were inserted via *Not*I / *Sgr*DI and *Pac*I / *Swa*I
pKG2-PIS	Derivative of pKG1-PIS in which hp4d-*hph*-*LIP2*t was exchanged for *URA3*p-*URA3*-*URA3*t via *Pci*I and *Sda*I
pACYC_assembly	Derivative of pACYC177 (New England Biolabs) in which a multiple cloning site comprising *Sda*I, *Apa*LI, *Nco*I, *Sal*I, *Acl*I, *Aat*II, *Ngo*MIV, *Avr*II, and *Pac*I restriction sites was inserted via *Dra*I and *Bam*HI
pACYC_BB1-4_C1_V1	Derivative of pACYC_assembly in which building block 1 of the synthetic *pfa* gene cluster version C1_V1 was inserted via *Sda*I and *Apa*LI, in which building block 2 of the synthetic *pfa* gene cluster version C1_V1 was inserted via *Apa*LI and *Acl*I, in which building block 3 of the synthetic *pfa* gene cluster version C1_V1 was inserted via *Acl*I and *Avr*II, and in which building block 4 of the synthetic *pfa* gene cluster version C1_V1 was inserted via *Avr*II and *Pac*I
pSynPfaPptAf2 (pAf2)	Derivative of pACYC_BB1-4_C1_V1 in which the vector backbone was exchanged for the vector backbone of pINA1312-*Sda*I-cm^R-*Pac*I via *Sda*I and *Pac*I
pSynPfaPptAf4 (pAf4)	Derivative of pSynPfaPptAf2 in which the vector backbone was exchanged for the vector backbone of pKG2-PIS via *Sda*I and *Pac*I
pACYC_BB1-3_C3	Derivative of pACYC_assembly in which building block 1 of the synthetic *pfa* gene cluster version C3 was inserted via *Sda*I and *Apa*LI, in which building block 2 of the synthetic *pfa* gene cluster version C3 was inserted via *Apa*LI and *Nco*I, and in which building block 3 of the synthetic *pfa* gene cluster version C3 was inserted via *Nco*I and *Acl*I
pSyn_BB1-6_C3	Derivative of pSynPfaPptAf2 in which building blocks 1+2 were exchanged for building blocks 1-3 of pACYC_BB1-3_C3 via *Sda*I and *Acl*I, in which the first part of building block 3 was exchanged for building block 4 of the synthetic *pfa* gene cluster version C3 via *Acl*I and *Kpn*I, in which the last part of building block 3 was exchanged for building block 5 of the synthetic *pfa* gene cluster version C3 via *Kpn*I and *Avr*II, and in which building block 4 was exchanged for building block 6 of the synthetic *pfa* gene cluster version C3 via *Avr*II and *Pac*I

Continuation of Supplementary Table 12.

Plasmid	Characteristics
pACYC_BB1-6_C3	Derivative of pSyn_BB1-6_C3 in which the vector backbone was exchanged for the vector backbone of pACYC_assembly via *Sda*I and *Pac*I
pSynPfaPptMr1 (pMr1)	Derivative of pSyn_BB1-6_C3 in which the vector backbone was exchanged for the vector backbone of pKG2-PIS via *Sda*I and *Pac*I
pACYC_BB1-6_C3_BB1+2+4+6_mod 5'	Derivative of pACYC_BB1-6_C3 in which building block 1 was exchanged for BB1_C3_mod 5' via *Sda*I and *Apa*LI, in which the 5' part of building block 2 was exchanged for BB2_C3_mod 5' via *Apa*LI and *Pci*I, in which the 5' part of building block 4 was exchanged for BB4_C3_mod 5' via *Acl*I and *Kpn*I, and in which building block 6 was exchanged for BB6_C3_mod 5' via *Avr*II and *Pac*I
pSynPfaPptMr2 (pMr2)	Derivative of pACYC_BB1-6_C3_BB1+2+4+6_mod 5' in which the vector backbone was exchanged for the vector backbone of pKG2-PIS via *Sda*I and *Pac*I
pACYC_SynHyb1	Derivative of pACYC_BB1-4_C1_V1 in which hp4d, the KS2, the CLF, the AT2, the DH2, and the DH3 domain of synthetic gene *pfa3* were replaced by the homologous domains of synthetic gene *pfa3* originating from *M. rosea* (SBNa008) via *Acl*I and *Fse*I
pSynHybPfaPpt1 (pHyb1)	Derivative of pSynHybPfaPpt2 in which the DH2, the DH3, and the AGPAT domain of synthetic gene *pfa3* were replaced by the homologous domains plus the DH4 domain of synthetic gene *pfa3* originating from *M. rosea* (SBNa008) via *Kpn*I and *Avr*II
pSynHybPfaPpt2 (pHyb2)	Derivative of pACYC_SynHyb1 in which the vector backbone was exchanged for the vector backbone of pKG2-PIS via *Sda*I and *Pac*I
pSynHybPfaPpt2a (pHyb2a)	Derivative of pSynHybPfaPpt2 in which the AGPAT domain of synthetic gene *pfa3* was replaced by the DH4 domain of synthetic gene *pfa3* from *M. rosea* (SBNa008) plus the AGPAT domain of synthetic gene *pfa3* originating from *A. fasciculatus* (SBSr002) via *Fse*I and *Avr*II
pSynHybPfaPpt5 (pHyb5)	Derivative of pSynPfaPptAf4 in which the DH2 and the DH3 domain of synthetic gene *pfa3* were replaced by the homologous domains of synthetic gene *pfa3* originating from *M. rosea* (SBNa008) via *Kpn*I and *Fse*I
pSynHybPfaPpt5a (pHyb5a)	Derivative of pSynHybPfaPpt5 in which the AGPAT domain of synthetic gene *pfa3* was replaced by the DH4 domain of synthetic gene *pfa3* from *M. rosea* (SBNa008) plus the AGPAT domain of synthetic gene *pfa3* originating from *A. fasciculatus* (SBSr002) via *Fse*I and *Avr*II
pSynHybPfaPpt6 (pHyb6)	Derivative of pSynPfaPptAf4 in which the AGPAT domain of synthetic gene *pfa3* was replaced by the DH4 domain plus the AGPAT domain of synthetic gene *pfa3* from *M. rosea* (SBNa008) via *Fse*I and *Avr*II
pSynHybPfaPpt6b (pHyb6b)	Derivative of pSynPfaPptAf4 in which the AGPAT domain of synthetic gene *pfa3* was replaced by the DH4 domain of synthetic gene *pfa3* from *M. rosea* (SBNa008) plus the AGPAT domain of synthetic gene *pfa3* originating from *A. fasciculatus* (SBSr002) via *Fse*I and *Avr*II
pSynHybPfaPpt6b-H2270A (pHyb6b-H2270A)	Derivative of pSynHybPfaPpt6b in which the DH4 and the AGPAT domain of synthetic gene *pfa3* were replaced by the identical domains, but carrying an active site H2270A point mutation within the DH4 domain, via *Fse*I and *Avr*II

Continuation of Supplementary Table 12.

Plasmid	Characteristics
pSynHybPfaPpt7 (pHyb7)	Derivative of pSynPfaPptAf4 in which synthetic genes *pfa2* and *pfa3* were replaced by synthetic genes *pfa2* and *pfa3* originating from *M. rosea* (SBNa008) via *Apa*LI and *Avr*II
pACYC_SynHyb3	Derivative of pACYC_BB1-4_C1_V1 in which synthetic gene *pfa2* was replaced by synthetic gene *pfa2* originating from *M. rosea* (SBNa008) via *Apa*LI and *Acl*I
pSynHybPfaPpt8 (pHyb8)	Derivative of pACYC_SynHyb3 in which the vector backbone was exchanged for the vector backbone of pKG2-PIS via *Sda*I and *Pac*I
pACYC_SynHyb4	Derivative of pACYC_BB1-4_C1_V1 in which the DH1 domain of synthetic gene *pfa2* was replaced by the PCR product of the homologous domain of synthetic gene *pfa2* originating from *M. rosea* (SBNa008) via *Kfl*I and *Acl*I
pSynHybPfaPpt9 (pHyb9)	Derivative of pACYC_SynHyb4 in which the vector backbone was exchanged for the vector backbone of pKG2-PIS via *Sda*I and *Pac*I
pACYC_SynHyb7	Derivative of pACYC_BB1-6_C3_BB1+2+4+6_mod 5' in which the KR and the DH1 domain of synthetic gene *pfa2* were replaced by the fusion PCR product consisting of the KR domain of synthetic gene *pfa2* originating from *M. rosea* (SBNa008) and the DH1 domain of synthetic gene *pfa2* originating from *A. fasciculatus* (SBSr002) via *Sal*I and *Acl*I
pSynHybPfaPpt15 (pHyb15)	Derivative of pACYC_SynHyb7 in which the vector backbone was exchanged for the vector backbone of pKG2-PIS via *Sda*I and *Pac*I
pACYC_BB1-4_C1_V2	Derivative of pACYC_assembly in which building block 1 of the synthetic *pfa* gene cluster version C1_V2 was inserted via *Sda*I and *Apa*LI, in which building block 2 of the synthetic *pfa* gene cluster version C1_V2 was inserted via *Apa*LI and *Acl*I, in which building block 3 of the synthetic *pfa* gene cluster version C1_V2 was inserted via *Acl*I and *Avr*II, and in which building block 4 of the synthetic *pfa* gene cluster version C1_V2 was inserted via *Avr*II and *Pac*I
pSynPfaPptAf7 (pAf7)	Derivative of pACYC_BB1-4_C1_V2 in which the vector backbone was exchanged for the vector backbone of pKG2-PIS via *Sda*I and *Pac*I
pACYC_BB1-4_C1_V1_*pfa2-mcherry*	Derivative of pACYC_BB1-4_C1_V1 in which the 3' end of synthetic gene *pfa2* was replaced by the 3' end of synthetic gene *pfa2* with fused *mcherry* coding sequence via *Kfl*I and *Acl*I
pACYC_BB1-4_C1_V2_*pfa2-mcherry*	Derivative of pACYC_BB1-4_C1_V2 in which the 3' end of synthetic gene *pfa2* was replaced by the 3' end of synthetic gene *pfa2* with fused *mcherry* coding sequence via *Kfl*I and *Acl*I
pACYC_BB1-4_C1_V1_*pfa3-mcherry*	Derivative of pACYC_BB1-4_C1_V1 in which the 3' end of synthetic gene *pfa3* was replaced by the 3' end of synthetic gene *pfa3* with fused *mcherry* coding sequence via *Pdi*I and *Avr*II
pSynPfaPptAf4-*pfa2-mcherry* (pAf4-*pfa2-mcherry*)	Derivative of pACYC_BB1-4_C1_V1_*pfa2-mcherry* in which the vector backbone was exchanged for the vector backbone of pKG2-PIS via *Sda*I and *Pac*I
pSynPfaPptAf7-*pfa2-mcherry* (pAf7-*pfa2-mcherry*)	Derivative of pACYC_BB1-4_C1_V2_*pfa2-mcherry* in which the vector backbone was exchanged for the vector backbone of pKG2-PIS via *Sda*I and *Pac*I

Continuation of Supplementary Table 12.

Plasmid	Characteristics
pSynPfaPptAf4-*pfa3-mcherry* (pAf4-*pfa3-mcherry*)	Derivative of pACYC_BB1-4_C1_V1_*pfa3-mcherry* in which the vector backbone was exchanged for the vector backbone of pKG2-PIS via *Sda*I and *Pac*I
pSynPfaPptAf7-*pfa3-mcherry* (pAf7-*pfa3-mcherry*)	Derivative of pSynPfaPptAf7 in which the 3' end of synthetic gene *pfa3* was replaced by the 3' end of synthetic gene *pfa3* with fused *mcherry* coding sequence via *Fse*I and *Avr*II

Supplementary Table 13: Expression strains constructed in this study.

Strain	Characteristics
Y. lipolytica Po1h::SynPfaPptAf2 clones (*Y. lipolytica* Po1h::Af2 clones)	*Y. lipolytica* Po1h with the “yeast cassette” of pSynPfaPptAf2 (liberated with *Not*I) randomly integrated in the genome
Y. lipolytica Po1h::SynPfaPptAf4 (*Y. lipolytica* Po1h::Af4)	*Y. lipolytica* Po1h with the “yeast cassette” of pSynPfaPptAf4 (liberated with *Swa*I and *Not*I) integrated into the preferred integration site (*YALI0_C05907g*) of the genome
Y. lipolytica Po1h::SynPfaPptMr1 (*Y. lipolytica* Po1h::Mr1)	*Y. lipolytica* Po1h with the “yeast cassette” of pSynPfaPptMr1 (liberated with *Swa*I and *Not*I) integrated into the preferred integration site (*YALI0_C05907g*) of the genome
Y. lipolytica Po1h::SynPfaPptMr2 (*Y. lipolytica* Po1h::Mr2)	*Y. lipolytica* Po1h with the “yeast cassette” of pSynPfaPptMr2 (liberated with *Swa*I and *Not*I) integrated into the preferred integration site (*YALI0_C05907g*) of the genome
Y. lipolytica Po1h::SynHybPfaPpt1 (*Y. lipolytica* Po1h::Hyb1)	*Y. lipolytica* Po1h with the “yeast cassette” of pSynHybPfaPpt1 (liberated with *Swa*I and *Not*I) integrated into the preferred integration site (*YALI0_C05907g*) of the genome
Y. lipolytica Po1h::SynHybPfaPpt2a (*Y. lipolytica* Po1h::Hyb2a)	*Y. lipolytica* Po1h with the “yeast cassette” of pSynHybPfaPpt2a (liberated with *Swa*I and *Not*I) integrated into the preferred integration site (*YALI0_C05907g*) of the genome
Y. lipolytica Po1h::SynHybPfaPpt5a (*Y. lipolytica* Po1h::Hyb5a)	*Y. lipolytica* Po1h with the “yeast cassette” of pSynHybPfaPpt5a (liberated with *Swa*I and *Not*I) integrated into the preferred integration site (*YALI0_C05907g*) of the genome
Y. lipolytica Po1h::SynHybPfaPpt6 (*Y. lipolytica* Po1h::Hyb6)	*Y. lipolytica* Po1h with the “yeast cassette” of pSynHybPfaPpt6 (liberated with *Swa*I and *Not*I) integrated into the preferred integration site (*YALI0_C05907g*) of the genome
Y. lipolytica Po1h::SynHybPfaPpt6b (*Y. lipolytica* Po1h::Hyb6b)	*Y. lipolytica* Po1h with the “yeast cassette” of pSynHybPfaPpt6b (liberated with *Swa*I and *Not*I) integrated into the preferred integration site (*YALI0_C05907g*) of the genome
Y. lipolytica Po1h::SynHybPfaPpt6b-H2270A (*Y. lipolytica* Po1h::Hyb6b-H2270A)	*Y. lipolytica* Po1h with the “yeast cassette” of pSynHybPfaPpt6b-H2270A (liberated with *Swa*I and *Not*I) integrated into the preferred integration site (*YALI0_C05907g*) of the genome
Y. lipolytica Po1h::SynHybPfaPpt7 (*Y. lipolytica* Po1h::Hyb7)	*Y. lipolytica* Po1h with the “yeast cassette” of pSynHybPfaPpt7 (liberated with *Swa*I and *Not*I) integrated into the preferred integration site (*YALI0_C05907g*) of the genome
Y. lipolytica Po1h::SynHybPfaPpt8 (*Y. lipolytica* Po1h::Hyb8)	*Y. lipolytica* Po1h with the “yeast cassette” of pSynHybPfaPpt8 (liberated with *Swa*I and *Not*I) integrated into the preferred integration site (*YALI0_C05907g*) of the genome
Y. lipolytica Po1h::SynHybPfaPpt9 (*Y. lipolytica* Po1h::Hyb9)	*Y. lipolytica* Po1h with the “yeast cassette” of pSynHybPfaPpt9 (liberated with *Swa*I and *Not*I) integrated into the preferred integration site (*YALI0_C05907g*) of the genome
Y. lipolytica Po1h::SynHybPfaPpt15 (*Y. lipolytica* Po1h::Hyb15)	*Y. lipolytica* Po1h with the “yeast cassette” of pSynHybPfaPpt15 (liberated with *Swa*I and *Not*I) integrated into the preferred integration site (*YALI0_C05907g*) of the genome
Y. lipolytica Po1h::SynPfaPptAf7 (*Y. lipolytica* Po1h::Af7)	*Y. lipolytica* Po1h with the “yeast cassette” of pSynPfaPptAf7 (liberated with *Swa*I and *Not*I) integrated into the preferred integration site (*YALI0_C05907g*) of the genome

Continuation of Supplementary Table 13.

Strain	Characteristics
Y. lipolytica Po1h::SynPfaPptAf4-*pfa2-mcherry* (*Y. lipolytica* Po1h::Af4-*pfa2-mcherry*)	*Y. lipolytica* Po1h with the "yeast cassette" of pSynPfaPptAf4-*pfa2-mcherry* (liberated with *Swa*I and *Not*I) integrated into the preferred integration site (*YALI0_C05907g*) of the genome
Y. lipolytica Po1h::SynPfaPptAf7-*pfa2-mcherry* (*Y. lipolytica* Po1h::Af7-*pfa2-mcherry*)	*Y. lipolytica* Po1h with the "yeast cassette" of pSynPfaPptAf7-*pfa2-mcherry* (liberated with *Swa*I and *Not*I) integrated into the preferred integration site (*YALI0_C05907g*) of the genome
Y. lipolytica Po1h::SynPfaPptAf4-*pfa3-mcherry* (*Y. lipolytica* Po1h::Af4-*pfa3-mcherry*)	*Y. lipolytica* Po1h with the "yeast cassette" of pSynPfaPptAf4-*pfa3-mcherry* (liberated with *Swa*I and *Not*I) integrated into the preferred integration site (*YALI0_C05907g*) of the genome
Y. lipolytica Po1h::SynPfaPptAf7-*pfa3-mcherry* (*Y. lipolytica* Po1h::Af7-*pfa3-mcherry*)	*Y. lipolytica* Po1h with the "yeast cassette" of pSynPfaPptAf7-*pfa3-mcherry* (liberated with *Swa*I and *Not*I) integrated into the preferred integration site (*YALI0_C05907g*) of the genome

Supplementary Table 14: Modified and subsequently normalized codon usage table of a subset of the *Yarrowia lipolytica* CLIB122 CDSs generated for the design of the artificial *pfa* gene cluster C1_V2. Codon counts (n) were obtained by omitting the first 20 and last 10 codons of the 1609 selected protein coding sequences, leading to synonymous codon fractions (f) and the formal relative adaptiveness values (w). 20 codons (shaded) bearing the lowest w' according to a reference codon table (Supplementary Table 13) generated from a reference set of predominantly ribosomal genes (Supplementary Table 19) were excluded, corresponding to a relative adaptiveness threshold of 0.04 regarding w'. The synonymous codon fractions were recalculated without the excluded codons (fn) before applying the codon usage table for sequence optimization. Relative adaptiveness values w' from the reference codon table were used for local CAI calculations. Orange and blue marks depict codons that are maximally enriched and depleted, respectively, in the reference set.

AA	Cod.	n	f	fn	w	AA	Cod.	n	f	fn	w
A (Ala)	GCC	31617	0.55	0.61	1	P (Pro)	CCC	20128	0.63	0.67	1
	GCT	20624	0.36	0.39	0.652		CCT	10125	0.32	0.33	0.503
	GCA	3000	0.05	0	0.095		CCG	860	0.03	0	0.043
	GCG	2113	0.04	0	0.067		CCA	765	0.02	0	0.038
C (Cys)	TGC	3914	0.55	0.55	1	Q (Gln)	CAG	22106	0.92	1	1
	TGT	3192	0.45	0.45	0.816		CAA	1819	0.08	0	0.082
D (Asp)	GAC	26588	0.69	0.69	1	R (Arg)	CGA	20693	0.78	0.92	1
	GAT	11740	0.31	0.31	0.442		CGG	2174	0.08	0	0.105
E (Glu)	GAG	36448	0.88	0.88	1		CGT	1760	0.07	0.08	0.085
	GAA	4771	0.12	0.12	0.131		AGA	1477	0.06	0	0.071
F (Phe)	TTC	17809	0.7	0.7	1		CGC	336	0.01	0	0.016
	TTT	7814	0.3	0.3	0.439		AGG	127	0	0	0.006
G (Gly)	GGC	17657	0.37	0.38	1	S (Ser)	TCC	18141	0.4	0.42	1
	GGT	16509	0.35	0.35	0.935		TCT	16772	0.37	0.39	0.925
	GGA	12844	0.27	0.27	0.727		TCG	5143	0.11	0.12	0.284
	GGG	537	0.01	0	0.03		AGC	3088	0.07	0.07	0.17
H (His)	CAC	9922	0.75	0.75	1		TCA	1432	0.03	0	0.079
	CAT	3342	0.25	0.25	0.337		AGT	1127	0.02	0	0.062
I (Ile)	ATC	19269	0.56	0.56	1	T (Thr)	ACC	23975	0.62	0.68	1
	ATT	15066	0.44	0.44	0.782		ACT	11311	0.29	0.32	0.472
	ATA	139	0	0	0.007		ACA	2197	0.06	0	0.092
K (Lys)	AAG	38631	0.94	1	1		ACG	1376	0.04	0	0.057
	AAA	2296	0.06	0	0.059	V (Val)	GTC	19676	0.43	0.44	1
L (Leu)	CTG	22138	0.43	0.46	1		GTG	12731	0.28	0.29	0.647
	CTC	17232	0.34	0.36	0.778		GTT	12232	0.27	0.27	0.622
	CTT	8705	0.17	0.18	0.393		GTA	895	0.02	0	0.045
	TTG	2072	0.04	0	0.094	W (Trp)	TGG	7647	1	1	1
	CTA	1147	0.02	0	0.052	Y (Tyr)	TAC	17858	0.9	0.9	1
	TTA	104	0	0	0.005		TAT	1988	0.1	0.1	0.111
M (Met)	ATG	11836	1	1	1	* (Ter)	TAA	1002	0.62	-	-
N (Asn)	AAC	24250	0.9	0.9	1		TAG	525	0.33	-	-
	AAT	2570	0.1	0.1	0.106		TGA	82	0.05	-	-

Supplementary Table 15: Codon usage table of predominantly ribosomal genes from *Yarrowia lipolytica* generated for the design of the artificial *pfa* gene cluster C1_V2. Codon counts (n) were obtained by omitting the first 20 and last 10 codons of the 92 selected protein coding sequences (Supplementary Table 17), leading to synonymous codon fractions (f) and the relative adaptiveness values (w). The latter were used for local CAI calculations of the artificial *pfa* gene cluster C1_V2. Codons with relative adaptiveness values up to 0.04 are shaded. For comparison purposes the gene copy number (GCN) for the matching tRNA is given. Note that base modification is assumed where matching tRNAs are missing (e.g., GCC:IGC and GCU:IGC for Ala). Orange and blue marks depict codons that are maximally enriched and depleted, respectively, compared to the genomic codon usage.

AA	Cod.	n	f	w	GCN	AA	Cod.	n	f	w	GCN
A (Ala)	GCC	853	0.61	1	0	P (Pro)	CCC	488	0.73	1	0
	GCT	505	0.36	0.592	30		CCT	168	0.25	0.344	21
	GCA	34	0.02	0.04	4		CCG	9	0.01	0.018	2
	GCG	14	0.01	0.016	2		CCA	7	0.01	0.014	3
C (Cys)	TGC	100	0.66	1	8	Q (Gln)	CAG	480	0.97	1	15
	TGT	51	0.34	0.51	0		CAA	16	0.03	0.033	3
D (Asp)	GAC	551	0.7	1	28	R (Arg)	CGA	1071	0.9	1	25
	GAT	237	0.3	0.43	0		CGT	47	0.04	0.044	1
E (Glu)	GAG	960	0.94	1	27		AGA	31	0.03	0.029	4
	GAA	57	0.06	0.059	6		CGG	27	0.02	0.025	0
F (Phe)	TTC	449	0.81	1	17		CGC	9	0.01	0.008	0
	TTT	106	0.19	0.236	0		AGG	0	0	0	1
G (Gly)	GGT	589	0.51	1	0	S (Ser)	TCC	450	0.49	1	0
	GGC	385	0.33	0.654	30		TCT	367	0.4	0.816	21
	GGA	175	0.15	0.297	11		AGC	43	0.05	0.096	6
	GGG	3	0	0.005	0		TCG	30	0.03	0.067	4
H (His)	CAC	283	0.83	1	12		AGT	13	0.01	0.029	0
	CAT	57	0.17	0.201	0		TCA	13	0.01	0.029	2
I (Ile)	ATC	600	0.68	1	0	T (Thr)	ACC	667	0.76	1	0
	ATT	274	0.31	0.457	26		ACT	183	0.21	0.274	22
	ATA	2	0	0.003	1		ACA	20	0.02	0.03	3
K (Lys)	AAG	1472	0.98	1	34		ACG	12	0.01	0.018	2
	AAA	29	0.02	0.02	4	V (Val)	GTC	698	0.54	1	0
L (Leu)	CTC	574	0.46	1	0		GTT	400	0.31	0.573	24
	CTG	412	0.33	0.718	13		GTG	183	0.14	0.262	8
	CTT	244	0.2	0.425	21		GTA	8	0.01	0.011	2
	TTG	12	0.01	0.021	3	W (Trp)	TGG	161	1	1	13
	CTA	7	0.01	0.012	2	Y (Tyr)	TAC	444	0.94	1	14
	TTA	2	0	0.003	1		TAT	28	0.06	0.063	0
M (Met)	ATG	253	1	1	18	* (Ter)	TAA	79	0.86	-	-
N (Asn)	AAC	588	0.95	1	16		TAG	11	0.12	-	-
	AAT	28	0.05	0.048	0		TGA	2	0.02	-	-

Supplementary Table 16: Codon usage table of predominantly ribosomal genes from *Aetherobacter fasciculatus* (SBSr002) generated for the design of the artificial *pfa* gene cluster C1_V2. Codon counts (n) were obtained by omitting the first 20 and last 10 codons of the 57 selected protein coding sequences (Supplementary Table 18), leading to synonymous codon fractions (f) and the relative adaptiveness values (w). The latter were used for local CAI calculations of the native *pfa* gene cluster. For comparison purposes the gene copy number (GCN) for the matching tRNA is given. Note that base modification is assumed where matching tRNAs are missing. Orange and blue marks depict codons that are formally enriched and depleted, respectively, compared to the genomic codon usage.

AA	Cod.	n	f	w	GCN	AA	Cod.	n	f	w	GCN
A (Ala)	GCC	551	0.49	1	2	P (Pro)	CCG	249	0.5	1	2
	GCG	434	0.39	0.788	2		CCC	227	0.45	0.912	2
	GCT	123	0.11	0.223	0		CCT	22	0.04	0.088	0
	GCA	18	0.02	0.033	2		CCA	2	0	0.008	1
C (Cys)	TGC	60	0.95	1	2	Q (Gln)	CAG	303	0.95	1	2
	TGT	3	0.05	0.05	0		CAA	17	0.05	0.056	2
D (Asp)	GAC	458	0.83	1	2	R (Arg)	CGC	639	0.78	1	0
	GAT	94	0.17	0.205	0		CGG	98	0.12	0.153	2
E (Glu)	GAG	625	0.85	1	2		CGT	70	0.08	0.11	2
	GAA	112	0.15	0.179	2		CGA	9	0.01	0.014	1
F (Phe)	TTC	308	0.98	1	1		AGG	8	0.01	0.013	1
	TTT	5	0.02	0.016	0		AGA	0	0	0.001	2
G (Gly)	GGC	722	0.74	1	3	S (Ser)	TCG	222	0.44	1	1
	GGG	123	0.13	0.17	2		AGC	177	0.35	0.797	1
	GGT	103	0.11	0.143	0		TCC	96	0.19	0.432	1
	GGA	24	0.02	0.033	2		TCT	6	0.01	0.027	0
H (His)	CAC	211	0.93	1	2		AGT	4	0.01	0.018	0
	CAT	15	0.07	0.071	0		TCA	1	0	0.005	1
I (Ile)	ATC	536	0.97	1	2	T (Thr)	ACG	292	0.5	1	2
	ATT	15	0.03	0.028	0		ACC	285	0.48	0.976	2
	ATA	0	0	0.001	1		ACT	9	0.02	0.031	0
K (Lys)	AAG	761	0.96	1	2		ACA	3	0.01	0.01	1
	AAA	34	0.04	0.045	2	V (Val)	GTC	596	0.63	1	2
L (Leu)	CTC	597	0.73	1	2		GTG	308	0.32	0.517	2
	CTG	186	0.23	0.312	1		GTT	41	0.04	0.069	0
	CTT	24	0.03	0.04	0		GTA	3	0	0.005	1
	TTG	13	0.02	0.022	2	W (Trp)	TGG	65	1	1	2
	CTA	1	0	0.002	2	Y (Tyr)	TAC	192	0.95	1	2
	TTA	0	0	0.001	1		TAT	10	0.05	0.052	0
M (Met)	ATG	201	1	1	2	* (Ter)	TGA	26	0.46	-	-
N (Asn)	AAC	246	0.97	1	2		TAG	23	0.4	-	-
	AAT	7	0.03	0.028	0		TAA	8	0.14	-	-

Supplementary Table 17: Formal codon bias of the reference gene subsets used for adaptation of the local CAI of genes *pfa1*, *pfa2*, *pfa3*, and *ppt* from the artificial *pfa* biosynthetic gene cluster C1_V2. DF denotes degrees of freedom (sum of the individual sizes of the synonymous codon groups, in each case subtracted by 1). N_{codons} are the codon counts of the ribosomal reference subsets and the automatically clustered larger subsets, respectively. G is the raw value from the G-statistic and ΔH is 50 % of the G-value (the log likelihood ratio), normalized by the observed codon count (N_{codons}) in the gene subsets[33]. ΔH values of the reference sets which were used for the optimization of the local CAI of cluster C1_V2 are underlined. A minimum length of 200 bp and offsets of 60 bp at the 5' ends as well as 30 bp at the 3' ends were applied to the evaluated coding sequences, respectively.

Gene set	DF	N_{codons}	G	p-value	ΔH[*)]
Aetherobacter fasciculatus (SBSr002) Ribo	41	10298	739.92	< .001	<u>0.036</u>
Aetherobacter fasciculatus (SBSr002) Auto	41	1309210	58247.04	<.001	0.022
Yarrowia lipolytica CLIB122 Ribo	41	15565	6368.89	< .001	<u>0.205</u>
Yarrowia lipolytica CLIB122 Auto	41	616372	103085.57	< .001	0.084

Supplementary Table 18: Reference set of predominantly ribosomal genes coding for hypothetically highly expressed proteins from *Aetherobacter fasciculatus* (SBSr002) (MSr9330). The initial collection of 64 sequences was reduced to 57 due to the applied length threshold of 200bp.

No.	Product	Locus_tag	No.	Product	Locus_tag
1	30S ribosomal protein S1	MSr9330_18190	30	50S ribosomal protein L9	MSr9330_17660
2	30S ribosomal protein S1	MSr9330_50480	31	50S ribosomal protein L10	MSr9330_46440
3	30S ribosomal protein S2	MSr9330_72100	32	50S ribosomal protein L11	MSr9330_46420
4	30S ribosomal protein S3	MSr9330_25510	33	50S ribosomal protein L13	MSr9330_22270
5	30S ribosomal protein S4	MSr9330_25710	34	50S ribosomal protein L14	MSr9330_25550
6	30S ribosomal protein S4	MSr9330_46070	35	50S ribosomal protein L15	MSr9330_25640
7	30S ribosomal protein S5	MSr9330_25620	36	50S ribosomal protein L16	MSr9330_25520
8	30S ribosomal protein S6	MSr9330_17680	37	50S ribosomal protein L17	MSr9330_25730
9	30S ribosomal protein S7	MSr9330_14540	38	50S ribosomal protein L18	MSr9330_25610
10	30S ribosomal protein S8	MSr9330_25590	39	50S ribosomal protein L19	MSr9330_88640
11	30S ribosomal protein S9	MSr9330_22260	40	50S ribosomal protein L20	MSr9330_39100
12	30S ribosomal protein S10	MSr9330_14510	41	50S ribosomal protein L21	MSr9330_22640
13	30S ribosomal protein S11	MSr9330_25700	42	50S ribosomal protein L22	MSr9330_25500
14	30S ribosomal protein S12	MSr9330_14550	43	50S ribosomal protein L23	MSr9330_14490
15	30S ribosomal protein S13	MSr9330_25690	44	50S ribosomal protein L24	MSr9330_25560
16	30S ribosomal protein S15	MSr9330_65250	45	50S ribosomal protein L25	MSr9330_17700
17	30S ribosomal protein S16	MSr9330_67560	46	50S ribosomal protein L27	MSr9330_22650
18	30S ribosomal protein S17	MSr9330_25540	47	50S ribosomal protein L28	MSr9330_67670
19	30S ribosomal protein S18	MSr9330_17670	48	50S ribosomal protein L29	MSr9330_25530
20	30S ribosomal protein S19	MSr9330_14470	49	50S ribosomal protein L30	MSr9330_25630
21	30S ribosomal protein S20	MSr9330_25150	50	50S ribosomal protein L31	MSr9330_27610
22	30S ribosomal protein S21	MSr9330_54870	51	elongation factor G	MSr9330_14530
23	50S ribosomal protein L1	MSr9330_46430	52	elongation factor G	MSr9330_42950
24	50S ribosomal protein L2	MSr9330_14480	53	elongation factor G	MSr9330_52660
25	50S ribosomal protein L3	MSr9330_48250	54	elongation factor G	MSr9330_89640
26	50S ribosomal protein L4	MSr9330_14500	55	elongation factor Ts	MSr9330_72110
27	50S ribosomal protein L5	MSr9330_25570	56	elongation factor Tu	MSr9330_14520
28	50S ribosomal protein L6	MSr9330_25600	57	elongation factor Tu	MSr9330_46370
29	50S ribosomal protein L7/L12	MSr9330_46450			

Supplementary Table 19: Reference set of predominantly ribosomal genes coding for hypothetically highly expressed proteins from *Yarrowia lipolytica* CLIB122. The initial collection of 97 sequences was reduced to 92 due to the applied length threshold of 200 bp.

No.	Product	Locus_tag	No.	Product	Locus_tag
1	40S ribosomal protein S0	YALI0A18205g	47	60S ribosomal protein L13	YALI0B12826g
2	40S ribosomal protein S1	YALI0F05676g	48	60S ribosomal protein L14	YALI0E00550g
3	40S ribosomal protein S2	YALI0E14465g	49	60S ribosomal protein L15	YALI0D24387g
4	40S ribosomal protein S3	YALI0E23694g	50	60S ribosomal protein L16	YALI0C09218g
5	40S ribosomal protein S4	YALI0D12903g	51	60S ribosomal protein L17	YALI0C15895g
6	40S ribosomal protein S5	YALI0F08569g	52	60S ribosomal protein L18	YALI0B08866g
7	40S ribosomal protein S6	YALI0F18766g	53	60S ribosomal protein L19	YALI0E25025g
8	40S ribosomal protein S7	YALI0A10725g	54	60S ribosomal protein L20	YALI0F24123g
9	40S ribosomal protein S8	YALI0F24959g	55	60S ribosomal protein L21	YALI0F05522g
10	40S ribosomal protein S9	YALI0F05544g	56	60S ribosomal protein L22	YALI0E32208g
11	40S ribosomal protein S11	YALI0F20482g	57	60S ribosomal protein L23	YALI0D10263g
12	40S ribosomal protein S12	YALI0F06160g	58	60S ribosomal protein L24	YALI0E23584g
13	40S ribosomal protein S13	YALI0F11055g	59	60S ribosomal protein L25	YALI0F25531g
14	40S ribosomal protein S14	YALI0D05753g	60	60S ribosomal protein L26	YALI0E21241g
15	40S ribosomal protein S15	YALI0F05803g	61	60S ribosomal protein L27	YALI0B05896g
16	40S ribosomal protein S16	YALI0B12848g	62	60S ribosomal protein L28	YALI0B21076g
17	40S ribosomal protein S17	YALI0F14465g	63	60S ribosomal protein L30	YALI0E23562g
18	40S ribosomal protein S18	YALI0D20614g	64	60S ribosomal protein L31	YALI0E24475g
19	40S ribosomal protein S19	YALI0B20504g	65	60S ribosomal protein L32	YALI0E30811g
20	40S ribosomal protein S20	YALI0E20581g	66	60S ribosomal protein L33	YALI0E31955g
21	40S ribosomal protein S21	YALI0D13794g	67	60S ribosomal protein L33	YALI0F31405g
22	40S ribosomal protein S22	YALI0D05731g	68	60S ribosomal protein L34	YALI0C05082g
23	40S ribosomal protein S22	YALI0F25399g	69	60S ribosomal protein L35	YALI0A09922g
24	40S ribosomal protein S23	YALI0A20746g	70	60S ribosomal protein L36	YALI0E30602g
25	40S ribosomal protein S24	YALI0D13728g	71	60S ribosomal protein L37	YALI0E01452g
26	40S ribosomal protein S25	YALI0C03872g	72	60S ribosomal protein L42	YALI0B04224g
27	40S ribosomal protein S26	YALI0E19701g	73	60S ribosomal protein L43	YALI0E34573g
28	40S ribosomal protein S27	YALI0E34826g	74	66S preribosome component MAK16	YALI0C08052g
29	40S ribosomal protein S28	YALI0C05148g	75	90S preribosome/SSU processome component KRR1	YALI0F30393g
30	60S acidic ribosomal protein P0	YALI0B14146g	76	acetate--CoA ligase	YALI0F05962g
31	60S acidic ribosomal protein P1	YALI0B12804g	77	ADP/ATP carrier protein	YALI0A10659g
32	60S acidic ribosomal protein P1	YALI0B21252g	78	ADP/ATP carrier protein	YALI0D08228g
33	60S acidic ribosomal protein P2	YALI0F05808g	79	ADP/ATP carrier protein	YALI0F19712g
34	60S ribosomal export protein NMD3	YALI0F08151g	80	AGC family protein serine/threonine kinase domain-containing protein	YALI0C15444g
35	60S ribosomal protein L1	YALI0E31911g	81	CBS/PB1 domain-containing protein	YALI0D18106g
36	60S ribosomal protein L2	YALI0F24739g	82	elongation factor 1 gamma domain-containing protein	YALI0B12562g
37	60S ribosomal protein L3	YALI0C21560g	83	elongation factor 1 gamma domain-containing protein	YALI0C24420g
38	60S ribosomal protein L4	YALI0C06820g	84	mRNA turnover protein MRT4	YALI0F31317g
39	60S ribosomal protein L5	YALI0E22352g	85	ribosome biogenesis protein BRX1	YALI0B11880g
40	60S ribosomal protein L6	YALI0E27830g	86	ribosome biogenesis protein NIP7	YALI0E34287g
41	60S ribosomal protein L7	YALI0E13618g	87	ribosome biogenesis protein NSA2	YALI0E10461g
42	60S ribosomal protein L8	YALI0F24695g	88	ribosome biogenesis protein RLP24	YALI0B09779g
43	60S ribosomal protein L9	YALI0E29073g	89	rRNA methyltransferase NOP1	YALI0C15873g
44	60S ribosomal protein L10	YALI0D13104g	90	small nucleolar ribonucleoprotein SNU13	YALI0F24497g
45	60S ribosomal protein L11	YALI0B15103g	91	ubiquitin-40S ribosomal protein S31 fusion protein	YALI0F09790g
46	60S ribosomal protein L12	YALI0D13882g	92	ubiquitin-60S ribosomal protein L40 fusion protein	YALI0F08745g

Supplementary References

1. Chaney, J. L., Steele, A., Carmichael, R., Rodriguez, A., Specht, A. T., Ngo, K., Li, J., Emrich, S. & Clark, P. L. Widespread position-specific conservation of synonymous rare codons within coding sequences. *PLoS Comput. Biol.* **13,** e1005531 (2017).
2. Qian, W., Yang, J. R., Pearson, N. M., Maclean, C. & Zhang, J. Balanced codon usage optimizes eukaryotic translational efficiency. *PLoS Genet.* **8,** e1002603 (2012).
3. Ikemura, T. Codon usage and tRNA content in unicellular and multicellular organisms. *Mol. Biol. Evol.* **2,** 13-34 (1985).
4. Sharp, P. M. & Li, W. H. The codon Adaptation Index--a measure of directional synonymous codon usage bias and its potential applications. *Nucleic acids Res.* **15,** 1281–1295 (1987).
5. Chu, D. et al. Translation elongation can control translation initiation on eukaryotic mRNAs. *EMBO J.* **33,** 21–34 (2014).
6. Pedersen, M. et al. The functional half-life of an mRNA depends on the ribosome spacing in an early coding region. *J. Mol. Biol.* **407,** 35–44 (2011).
7. Shirokikh, N. E. & Preiss, T. Translation initiation by cap-dependent ribosome recruitment: Recent insights and open questions. *Wiley Interdiscip. Rev. RNA* **9,** e1473 (2018).
8. Dvir, S. et.al. Deciphering the rules by which 5′-UTR sequences affect protein expression in yeast. *Proc. Natl. Acad. Sci. USA* **110,** E2792–E2801 (2013).
9. Shah, P., Ding, Y., Niemczyk, M., Kudla, G. & Plotkin, J. B. Rate-limiting steps in yeast protein translation. *Cell* **153,** 1589–1601 (2013).
10. Lorenz, R. et al. ViennaRNA Package 2.0. *Algorithms Mol. Biol.* **6,** 26 (2011).
11. Komar, A. A. A pause for thought along the co-translational folding pathway. *Trends Biochem. Sci.* **34,** 16–24 (2009).
12. O'Brien, E. P., Vendruscolo, M. & Dobson, C. M. Kinetic modelling indicates that fast-translating codons can coordinate cotranslational protein folding by avoiding misfolded intermediates. *Nat. Commun.* **5,** 2988 (2014).
13. Angov, E., Hillier, C. J., Kincaid, R. L. & Lyon, J. A. Heterologous protein expression is enhanced by harmonizing the codon usage frequencies of the target gene with those of the expression host. *PloS One* **3,** e2189 (2008).
14. Buhr, F. et al. Synonymous Codons Direct Cotranslational Folding toward Different Protein Conformations. *Mol. Cell* **61,** 341–351 (2016).
15. Zhang, G., Hubalewska, M. & Ignatova, Z. Transient ribosomal attenuation coordinates protein synthesis and co-translational folding. *Nat. Struct. Mol. Biol.* **16,** 274 (2009).
16. Sander, I. M., Chaney, J. L. & Clark, P. L. Expanding Anfinsen's principle: contributions of synonymous codon selection to rational protein design. *J. Am. Chem. Soc.* **136,** 858–861 (2014).
17. Navon, S. & Pilpel, Y. The role of codon selection in regulation of translation efficiency deduced from synthetic libraries. *Genome Biol.* **12,** R12 (2011).
18. Pershing, N. L. et al. Rare codons capacitate Kras-driven de novo tumorigenesis. *J. Clin. Invest.* **125,** 222–233 (2015).

19. Spencer, P. S., Siller, E., Anderson, J. F. & Barral, J. M. Silent substitutions predictably alter translation elongation rates and protein folding efficiencies. *J. Mol. Biol.* **422,** 328–335 (2012).
20. Brockmann, R., Beyer, A., Heinisch, J. J. & Wilhelm, T. Posttranscriptional expression regulation: what determines translation rates? *PLoS Comput. Biol.* **3,** e57 (2007).
21. Weinberg, D. E. et al. Improved Ribosome-Footprint and mRNA Measurements Provide Insights into Dynamics and Regulation of Yeast Translation. *Cell Rep.* **14,** 1787–1799 (2016).
22. Gardin, J. et al. Measurement of average decoding rates of the 61 sense codons in vivo. *Elife* **3,** e03735 (2014).
23. Villada, J. C., Brustolini, O. J. B. & Batista da Silveira, W. Integrated analysis of individual codon contribution to protein biosynthesis reveals a new approach to improving the basis of rational gene design. *DNA Res.* **24,** 419–434 (2017).
24. Ran, W. & Higgs, P. G. Contributions of speed and accuracy to translational selection in bacteria. *PloS One* **7,** e51652 (2012).
25. Burke, E. K. & Bykov, Y. The late acceptance Hill-Climbing heuristic. *European Journal of Operational Research* **258,** 70–78 (2017).
26. Wen, J. D. et al. Following translation by single ribosomes one codon at a time. *Nature* **452,** 598 (2008).
27. Neuveglise-Degouy, C., Marck, C. & Gaillardin, C. The intronome of budding yeasts. *C. R. Biol.* **334,** 662–670 (2011).
28. Seligmann, H. & Pollock, D. D. The ambush hypothesis: hidden stop codons prevent off-frame gene reading. *DNA Cell Biol.* **23,** 701–705 (2004).
29. Noé, L. & Kucherov, G. YASS: enhancing the sensitivity of DNA similarity search. *Nucleic acids Res.* **33,** W540–W543 (2005).
30. Anikin, M., Molodtsov, V., Temiakov, D. & McAllister, W. T. *Transcript Slippage and Recoding. In Recoding: Expansion of Decoding Rules Enriches Gene Expression* (Springer, New York, 2010).
31. Reis, M. D., Savva, R. & Wernisch, L. Solving the riddle of codon usage preferences: a test for translational selection. *Nucleic acids Res.* **32,** 5036–5044 (2004).
32. Tuller, T. et al. An evolutionarily conserved mechanism for controlling the efficiency of protein translation. *Cell* **141,** 344–354 (2010).
33. Waldman, Y. Y., Tuller, T., Shlomi, T., Sharan, R. & Ruppin, E. Translation efficiency in humans: tissue specificity, global optimization and differences between developmental stages. *Nucleic acids Res.* **38,** 2964–2974 (2010).
34. Grosjean, H., de Crécy-Lagard, V. & Marck, C. Deciphering synonymous codons in the three domains of life: co-evolution with specific tRNA modification enzymes. *FEBS Lett.* **584,** 252–264 (2010).
35. Percudani, R., Pavesi, A. & Ottonello, S. Transfer RNA gene redundancy and translational selection in *Saccharomyces cerevisiae*. *J. Mol. Biol.* **268,** 322–330 (1997).
36. Novoa, E. M., Pavon-Eternod, M., Pan, T. & de Pouplana, L. R. A role for tRNA modifications in genome structure and codon usage. *Cell* **149,** 202–213 (2012).
37. Diwan, G. D. & Agashe, D. Wobbling Forth and Drifting Back: The Evolutionary History and Impact of Bacterial tRNA Modifications. *Mol. Biol. Evol.* **35,** 2046–2059 (2018).

38. Chan, P. P. & Lowe, T. M. GtRNAdb 2.0: an expanded database of transfer RNA genes identified in complete and draft genomes. *Nucleic acids Res.* **44,** D184–D189 (2015).
39. Lowe, T. M. & Chan, P. P. tRNAscan-SE On-line: integrating search and context for analysis of transfer RNA genes. *Nucleic acids Res.* **44,** W54–W57 (2016).
40. Weenink, T., McKiernan, R. M. & Ellis, T. Rational Design of RNA Structures that Predictably Tune Eukaryotic Gene Expression. *BioRxiv* 137877 (2017).
41. Davidow, L. S. et al. The *Yarrowia lipolytica LEU2* gene. *Curr. Genet.* **11,** 377–383 (1987).
42. Nicaud, J.M. et al. Protein expression and secretion in the yeast *Yarrowia lipolytica*. *FEMS Yeast Res.* **2,** 371–379 (2002).
